# Supplementary material for: Revealing mitf functions and visualizing allografted tumor metastasis in colorless and immunodeficient Xenopus tropicalis
Source: Commun Biol. 2024 Mar 5;7:275. doi: 10.1038/s42003-024-05967-3 (PMC10915148; doi:10.1038/s42003-024-05967-3)
Supplement: Supplementary file 2 — Supplementary Information [file 42003_2024_5967_MOESM2_ESM.pdf]

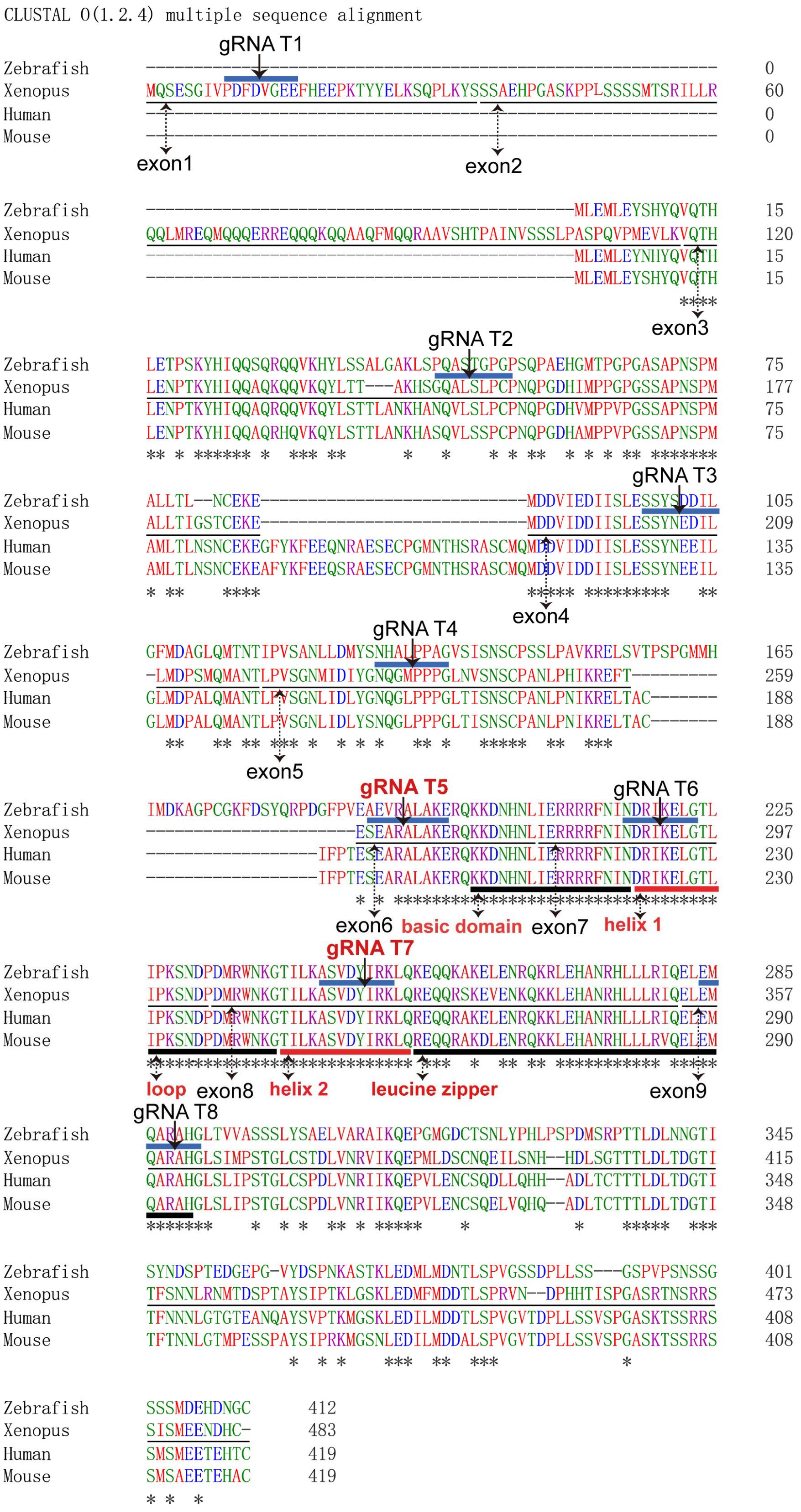

Supplementary Figure 1. Multiple sequence alignment results of Mitf protein between different species (using the online tool Clustal Omega) are shown. The positions of each gRNA, exons encoding amino acids, and multiple conserved structural domains are marked in the diagram. "*" represents highly conserved amino acids.

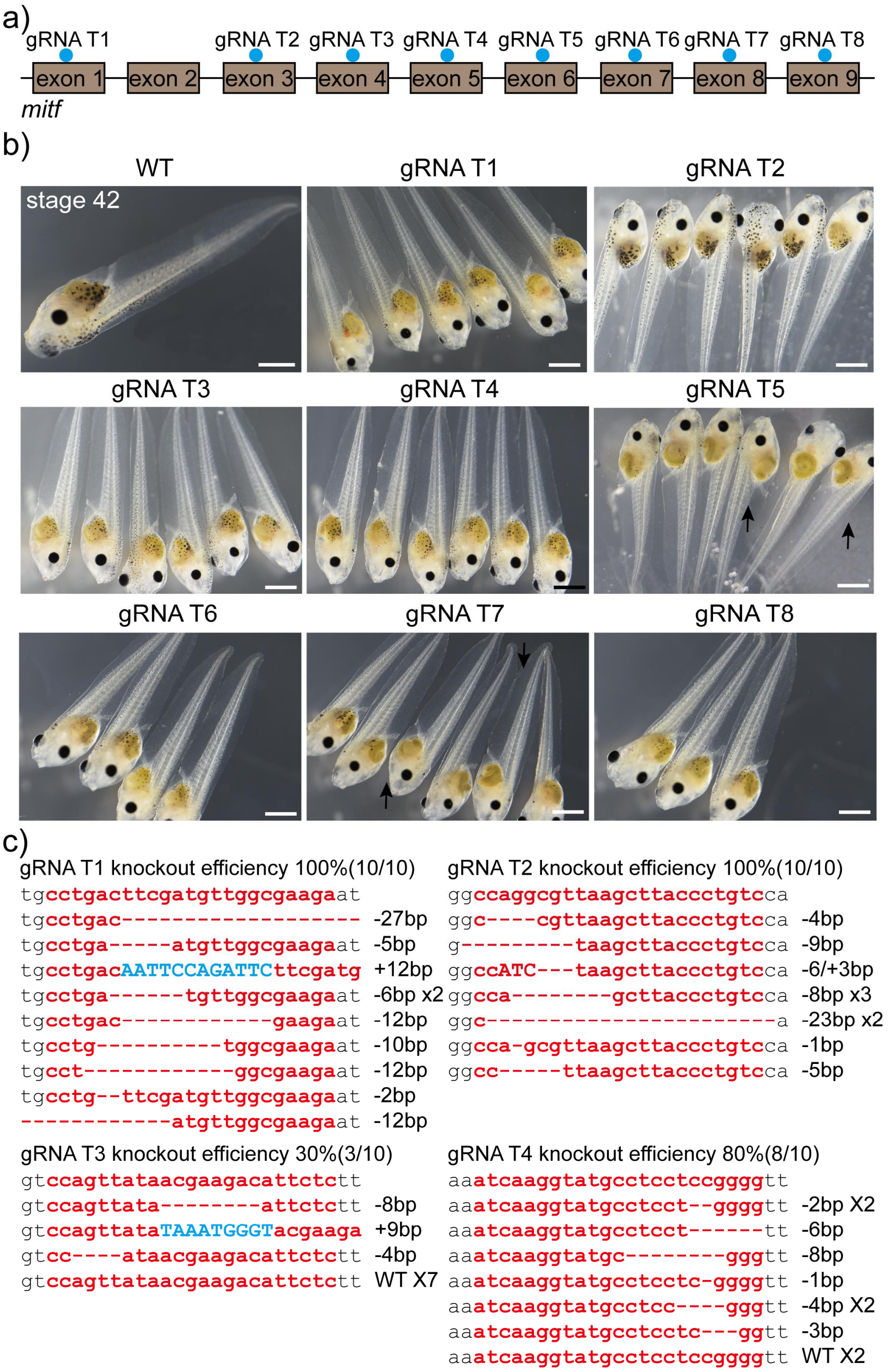

Supplementary Figure 2. Knock out *mitf* in *Xenopus tropicalis*. (a) For knocking out *mitf* in *Xenopus tropicalis*, eight guide RNAs were tailored to specifically target eight distinct exons of *mitf*. **(b)** After employing the CRISPR/Cas9 system to target the *mitf* gene using eight guide RNAs, some founder generation (G0) tadpoles at stage 42 exhibited a phenotype characterized by the loss of melanophores, as indicated by the black arrows. After conducting the experiment three times independently, a comparable phenotype was consistently observed. **(c)** The results of Sanger sequencing indicating the knockout efficiency of gRNAs T1 to T4 were presented. The gRNA target sequence was represented by red letters, the insertion sequence by blue capital letters, and base deletions by red dashed lines. The scale bar in **(b)** is 1 mm.

a)

gRNA T5 knockout efficiency 70%(7/10)

```
tcggaggcaagagcgttggccaaggaa
tcg---------------------gaa  -21bp
tcggaggcaagagcg----AAaaggaa  -6/+2bp
tcggaggcaagagcgttgg-Aaaggaa  -2/+1bp
tcggaggcaagagcgttgg------aa  -6bp x2
tcggaggcaag-------------gaa  -13bp
tcggaggcaagagcgttT-ccaaggaa  -10bp
tcggaggcaagagcgttggccaaggaa  WT X3
```

gRNA T6 knockout efficiency 100%(10/10)

```
tcaatgatcgcattaaagaattaggaa
tcaatgatcgcattaaagaa----gaa  -4bp
tcaatgatcgcattaaagaatt-ggaa  -1bp X2
tcaatgatcgcatt---------ggaa  -9bp
tcaatgatcgcattTT-------ggaa  -9/+2bp
t----------------------ggaa  -22bp
tcaatgatcgcattaaa-aattaggaa  -1bp X2
tcaatgatcgcattaaa-----aggaa  -5bp
tcaatgatcgcattaaA----taggaa  -5/+1bp
```

gRNA T7 knockout efficiency 80%(8/10)

```
agcctcggtggattacattcgcaaact
agcctcgg-ggattacattcgcaaact  -1bp
agcctcgg---attacattcgcaaact  -3bp X5
ag---------attacattcgcaaact  -9bp X2
agcctcggtggattacattcgcaaact  WT X2
```

gRNA T8 knockout efficiency 20%(2/10)

```
tagagatgcaagctcgcgcacatggcc
tagagatgcaagctcgcgc-------c  -7bp
tagagatgcaagctcgc---------c  -9bp
tagagatgcaagctcgcgcacatggcc  WT X8
```

b)

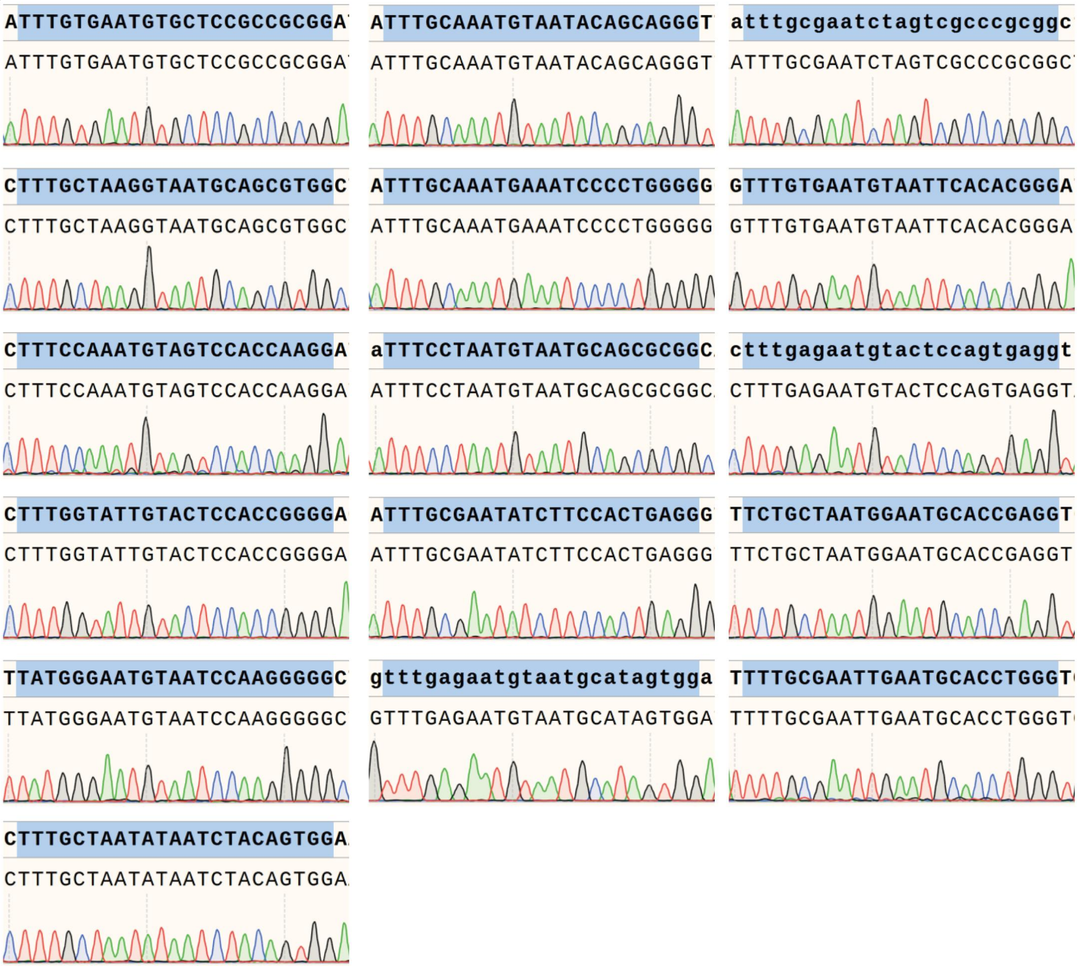

Supplementary Figure 3. The incidence of off-target effects in the disruption of *Xenopus tropicalis mitf* using CRISPR/Cas9 is relatively low. (a) The results of Sanger sequencing indicating the knockout efficiency of gRNAs T5 to T8 were presented. The gRNA target sequence was represented by red letters, the insertion sequence by blue capital letters, and base deletions by red dashed lines. **(b)** The outcomes of Sanger sequencing for off-target sites with up to four mismatches were presented.

a)

parent: G0 founder
offsprings
with melanophores: 46% (56/123)
without melanophores: 54% (67/123)

parent: F1 heterozygote
offsprings
with melanophores: 74% (99/134)
without melanophores: 26% (35/134)

b)

heterozygote genotype of F1
allele 1
agcctcggtggattacattcgcaaact
allele 2
agcct-------ttacattcgcaaact -7bp X3
------------ttacattcgcaaact -23bp X1
agcctcgg---attacattcgcaaact -3bp X2
agcct---t---ttacattcgcaaact -6bp X1

c)

compound homozygote genotype of F1
allele 1
agcct-------ttacattcgcaaact -7bp
agcctcggtTGCgattacattcgcaaa -1/+3bp
agcctcgg---attacattcgcaaact -3bp
agcct---t---ttacattcgcaaact -6bp
allele 2
agcctc----gattacattcgcaaact -4bp
agcctcggtTGCgattacattcgcaaa -1/+3bp
ag-----A-----tacattcgcaaact -11/+1bp
-----------------ttcgcaaact -19bp
agcct--()-gattacattcgcaaact -5/+107bp
(GCCGGGGGATGTTGGGTAGGGGGTTCCTCAC
GGTGAGGGCAGTGAGGGGGTTGGGGAATGCCC
TGCCGGGGGATGTTGGGTAGGGGGTTCCTCAC
GGTGAGGGCAGT)

Type 1: 2(-7, -4),2(-1/+3, -19),1(-1/+3, -1/+3)
Type 2: 2(-7, -5/+107),5(-6, -19),1(-3, -11/+1)
1(-6, -4)
Type 3: 1(-6, -5/+107)

d)

compound homozygote genotype of F2
allele 1
agcctc----gattacattcgcaaact -4bp
-----------------ttcgcaaact -19bp
allele 2
agcct-------ttacattcgcaaact -7bp
agcct--()-gattacattcgcaaact -5/+107bp
(GCCGGGGGATGTTGGGTAGGGGGTTCCTCAC
GGTGAGGGCAGTGAGGGGGTTGGGGAATGCCC
TGCCGGGGGATGTTGGGTAGGGGGTTCCTCAC
GGTGAGGGCAGT)

Type 1: 4(-19, -7),3(-4, -7)
Type 2: 6(-4, -5/+107),5(-19, -5/+107)

Supplementary Figure 4. The transmission efficiency of germline and genotypes of offspring of *mitf* knockout *Xenopus tropicalis* were shown. **(a)** The proportion of melanophore-bearing offspring resulting from a random G0 pair and a random F1 pair of frogs were presented. **(b)** The heterozygote genotype of F1 was shown. Seven tadpoles resulting from assortative mating of the randomly selected G0 pair in panel **(a)** were selected for genotyping. **(c)** The homozygous genotype for the F1 compound was depicted, and the corresponding genotype frequencies were presented at the bottom of Panel **(c)**. 15 tadpoles resulting from assortative mating of the randomly selected G0 pair in panel **(a)** were selected for genotyping. **(d)** The homozygous genotype for the F2 compound was shown, and the corresponding genotype frequencies was portrayed at the bottom of Panel **(d)**. To genotype the offspring, 18 tadpoles were chosen as a result of assortative mating of the randomly selected F1 pair in panel **(a)**. The gRNA target sequence was represented by red letters, the insertion sequence by blue capital letters, and base deletions by red dashed lines.

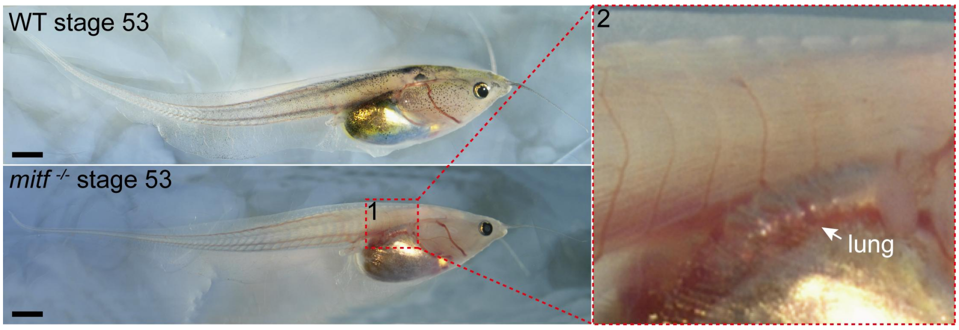

Supplementary Figure 5. Lateral view of stage 53 WT and F1 *mitf*$^{-/-}$ *Xenopus tropicalis* tadpoles. The F1 *mitf*$^{-/-}$ *Xenopus tropicalis* tadpole exhibited melanophores loss throughout the entire body at stages 53 compared to wild type tadpoles from the same batch. The development of the internal organ, the lung, was clearly visible. Red dashed box**2** corresponded to magnified view of red dashed box**1**. Scale bar is 1 mm.

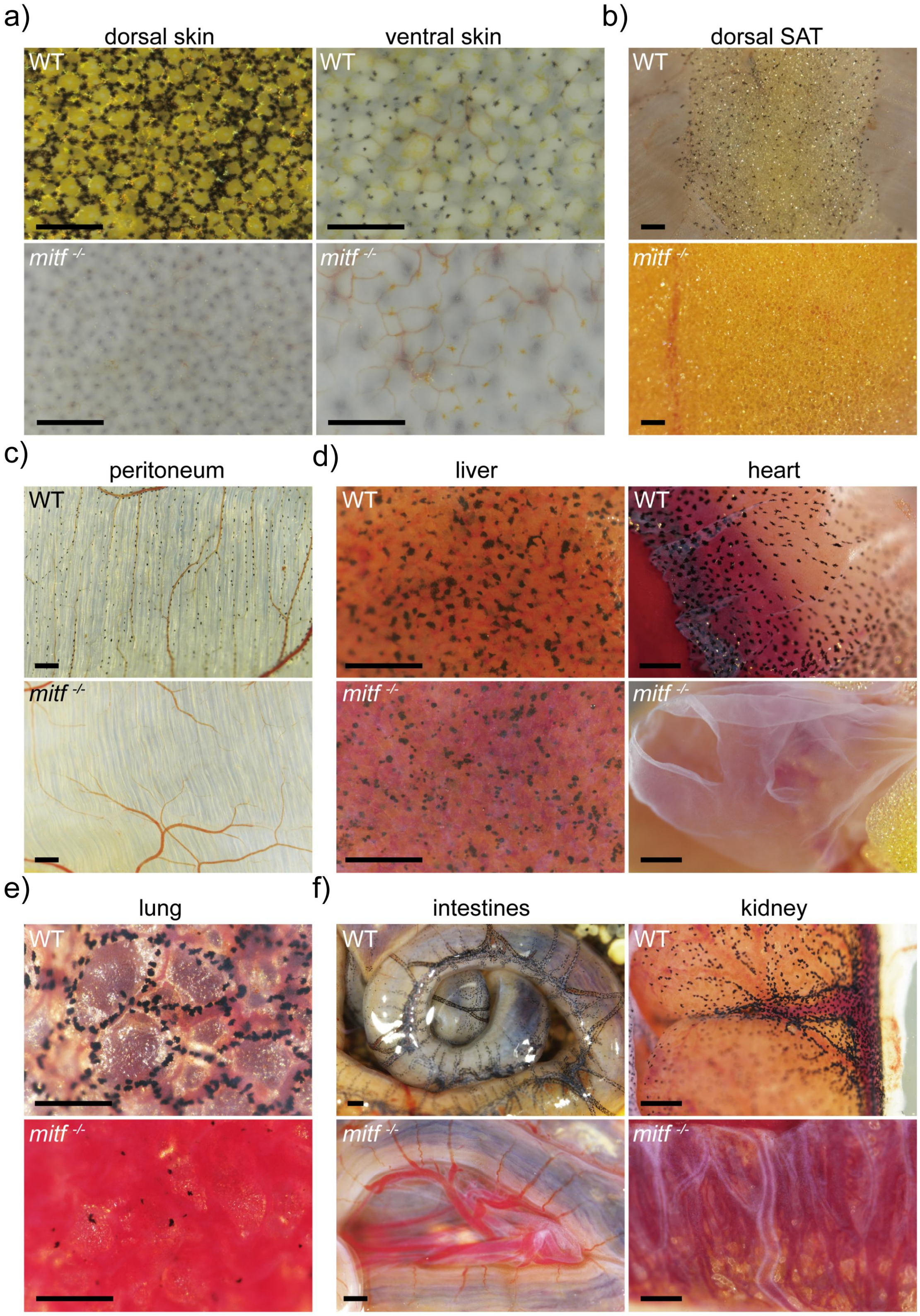

SAT： subcutaneous adipose tissue

Supplementary Figure 6. The melanin pigmentation of one-year-old *Xenopus tropicalis* was compared between WT and F1 *mitf*$^{-/-}$ frogs. **(a)-(f)** The comparison of the melanin pigmentation patterns in multiple body regions and various internal organs of WT and *mitf*$^{-/-}$ *Xenopus tropicalis* frogs was depicted. Three WT and three *mitf*$^{-/-}$ *Xenopus tropicalis* were observed, respectively. Each scale bar is 0.5 mm.

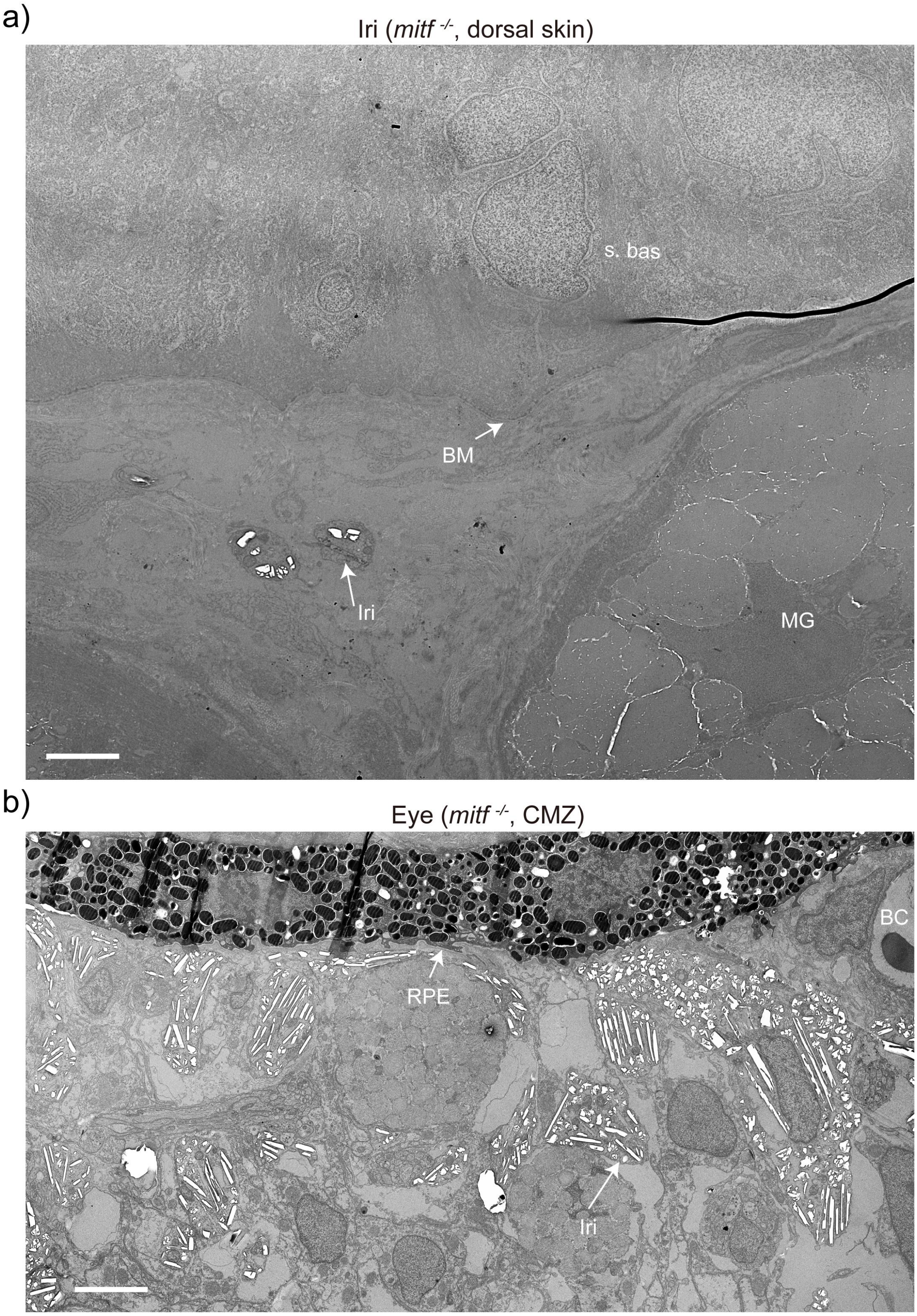

Supplementary Figure 7. (a) The TEM examination of the dorsal skin of 1-year-old *mitf*$^{-/-}$ *Xenopus tropicalis* was exhibited. **(b)** The TEM examination of the ciliary marginal zone in retina of 1-year-old *mitf*$^{-/-}$ *Xenopus tropicalis* was exhibited. **Mel**, melanophore; **Xan**, xanthophore; **Iri**, iridophore; **BM**, basement membrane; **MG**, mucous gland; **GG**, granular gland; **s.bas**, stratum basale; **BC**, blood cell; **CMZ**, ciliary marginal zone. The scale bar is 2 μm.

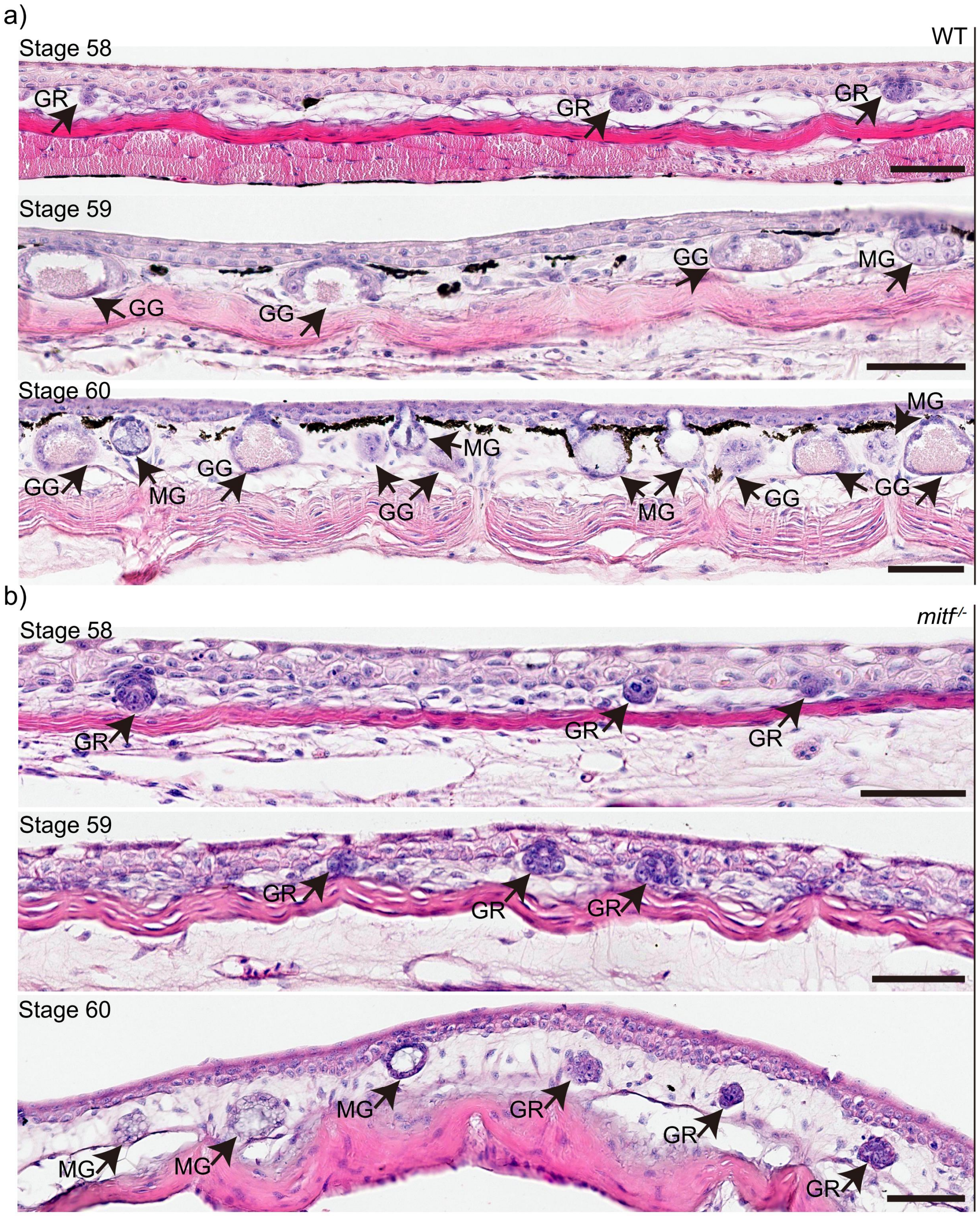

Supplementary Figure 8. Development of glands in WT and *mitf$^{-/-}$ Xenopus tropicalis*. (a) The development of glands in WT frogs from stage 58 to stage 60 was illustrated. **(b)** The development of glands in *mitf$^{-/-}$* frogs from stage 58 to stage 60 was delineated. **GG**, granular gland; **GR**, gland rudiment; **MG**, mucous gland. The scale is 60 μm.

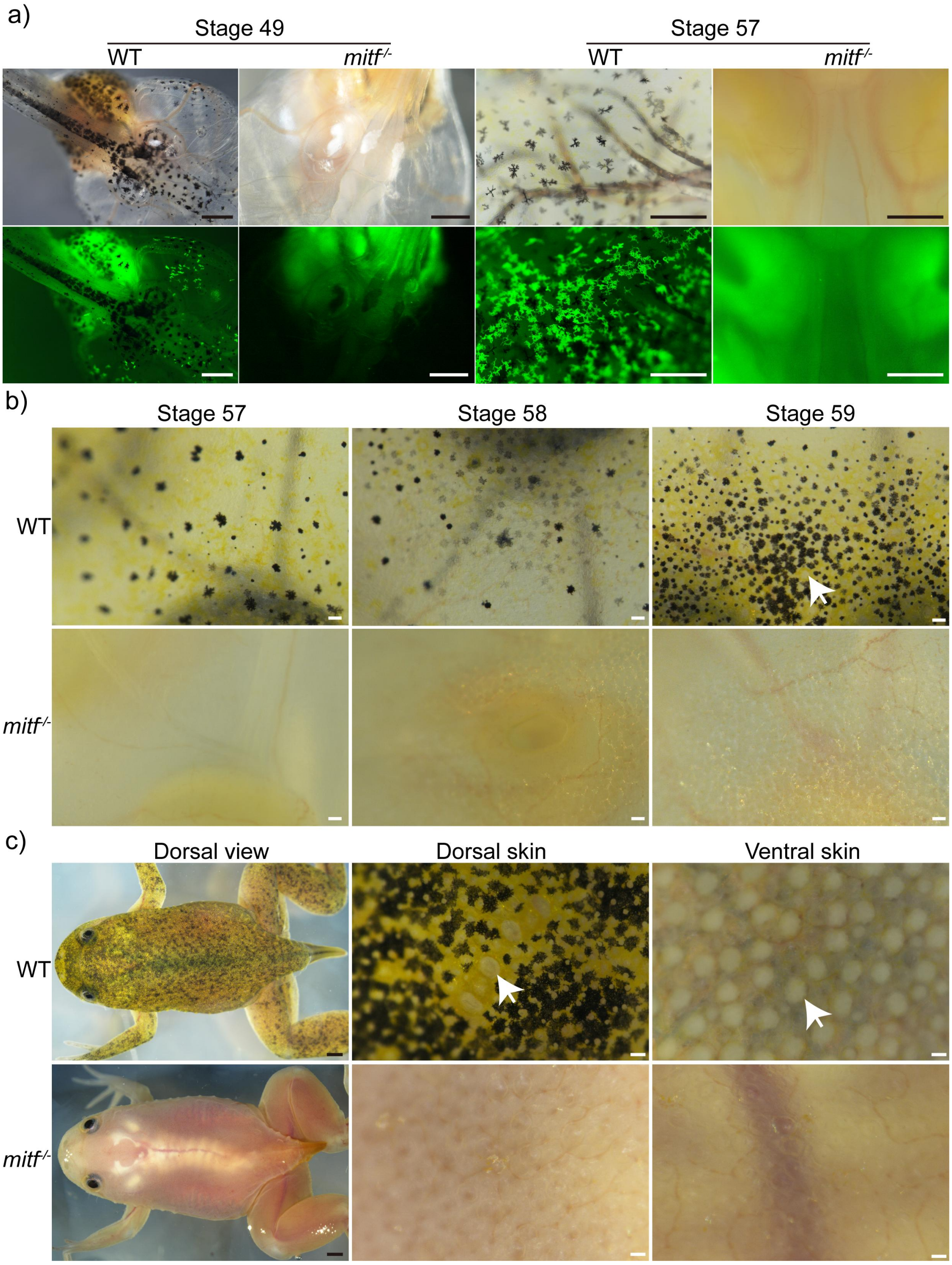

Supplementary Figure 9. Xanthophores and granular glands were notably absent in the skin of *mitf*$^{-/-}$ *Xenopus tropicalis*. **(a)** The intrinsic green fluorescence indicative of xanthophores was not observed in stage 49 and stage 57 *mitf*$^{-/-}$ *Xenopus tropicalis*. **(b)** The progression of granular gland development from stage 57 to stage 59 in both WT and *mitf*$^{-/-}$ *Xenopus tropicalis*, with white arrows denoting the emergence of developing granular glands. **(c)** The development of fully matured granular glands on the skin of WT and *mitf*$^{-/-}$ *Xenopus tropicalis* nearing completion of metamorphosis, with white arrows indicating well developed granular glands. **(a)**, the scale bar is 0.5 mm. **(b)**, the scale bar is 50 μm. For the photographs of whole frogs in **(c)**, the scale bar is 1 mm, while the scale bar in the images of skin is 50 μm.

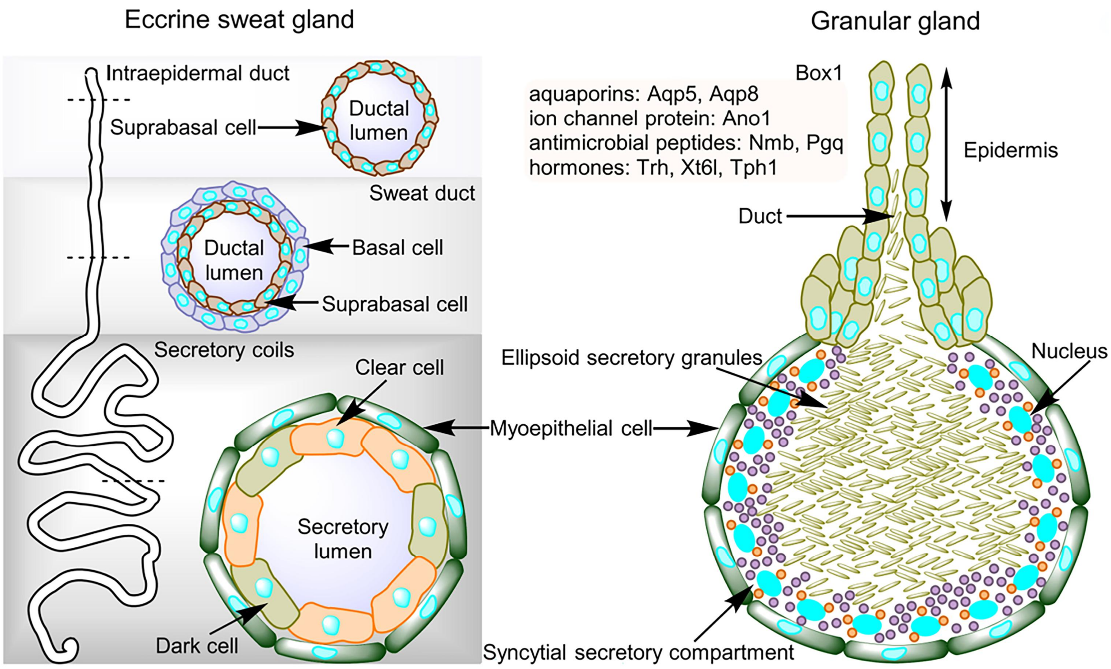

Supplementary Figure 10. Similarities and differences between the eccrine sweat gland of mouse and the granular gland of *Xenopus tropicalis*. Box1 displays the proteins co-expressed in the eccrine sweat gland of mice and the granular gland of *Xenopus tropicalis*. The schematic diagram of the eccrine sweat gland of mouse, with the cross-sectional diagram corresponding to the dotted lines, is presented on the right side.

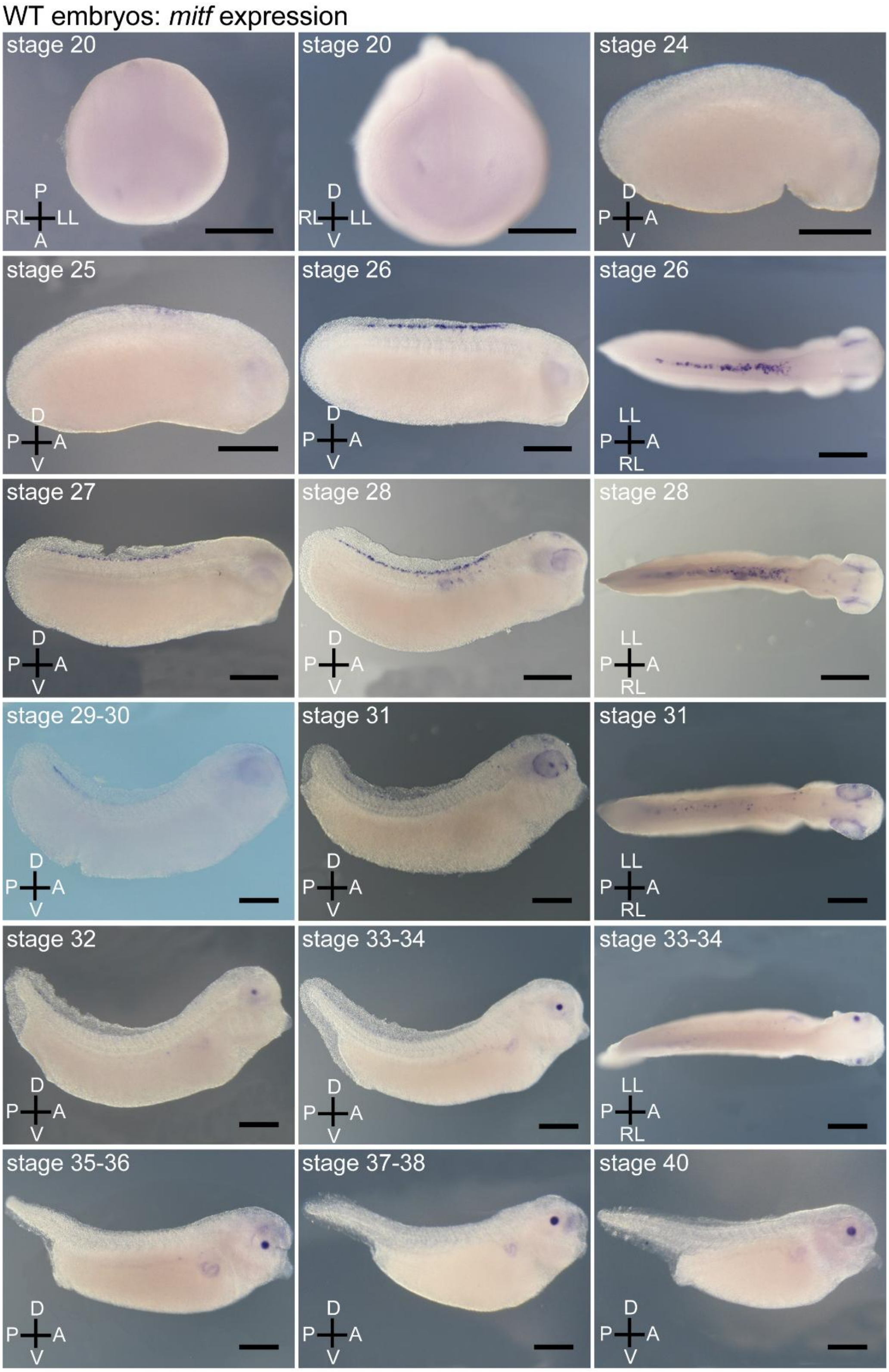

Supplementary Figure 11. The WISH results of *mitf* mRNA expression during embryonic development of WT *Xenopus tropicalis*. From stage 20 to stage 40, approximately 6-10 embryos per stage were used for in situ hybridization detection of *mitf* mRNA. **RL** represents right lateral view, **LL** represents left lateral view, **D** represents dorsal view, **V** represents ventral view, **P** represents posterior view, and **A** represents anterior view. Each scale bar is 0.5 mm.

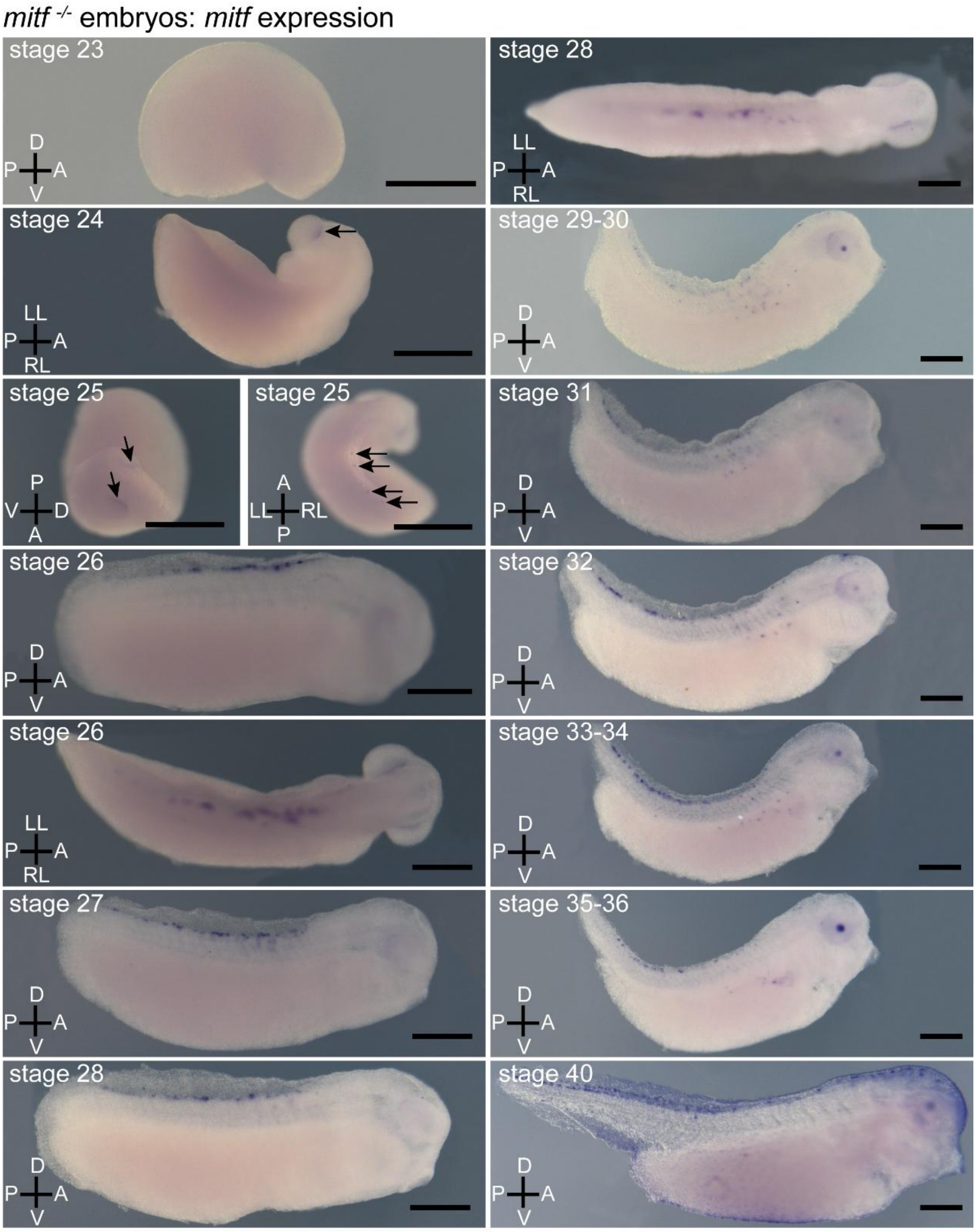

Supplementary Figure 12. The WISH results of *mitf* mRNA expression during embryonic development of *mitf*$^{-/-}$ *Xenopus tropicalis*. From stage 23 to stage 40, approximately 6-10 embryos per stage were used for in situ hybridization detection of *mitf* mRNA. **RL** represents right lateral view, **LL** represents left lateral view, **D** represents dorsal view, **V** represents ventral view, **P** represents posterior view, and **A** represents anterior view. Black arrow indicates the weaker signals. Each scale bar is 0.5 mm.

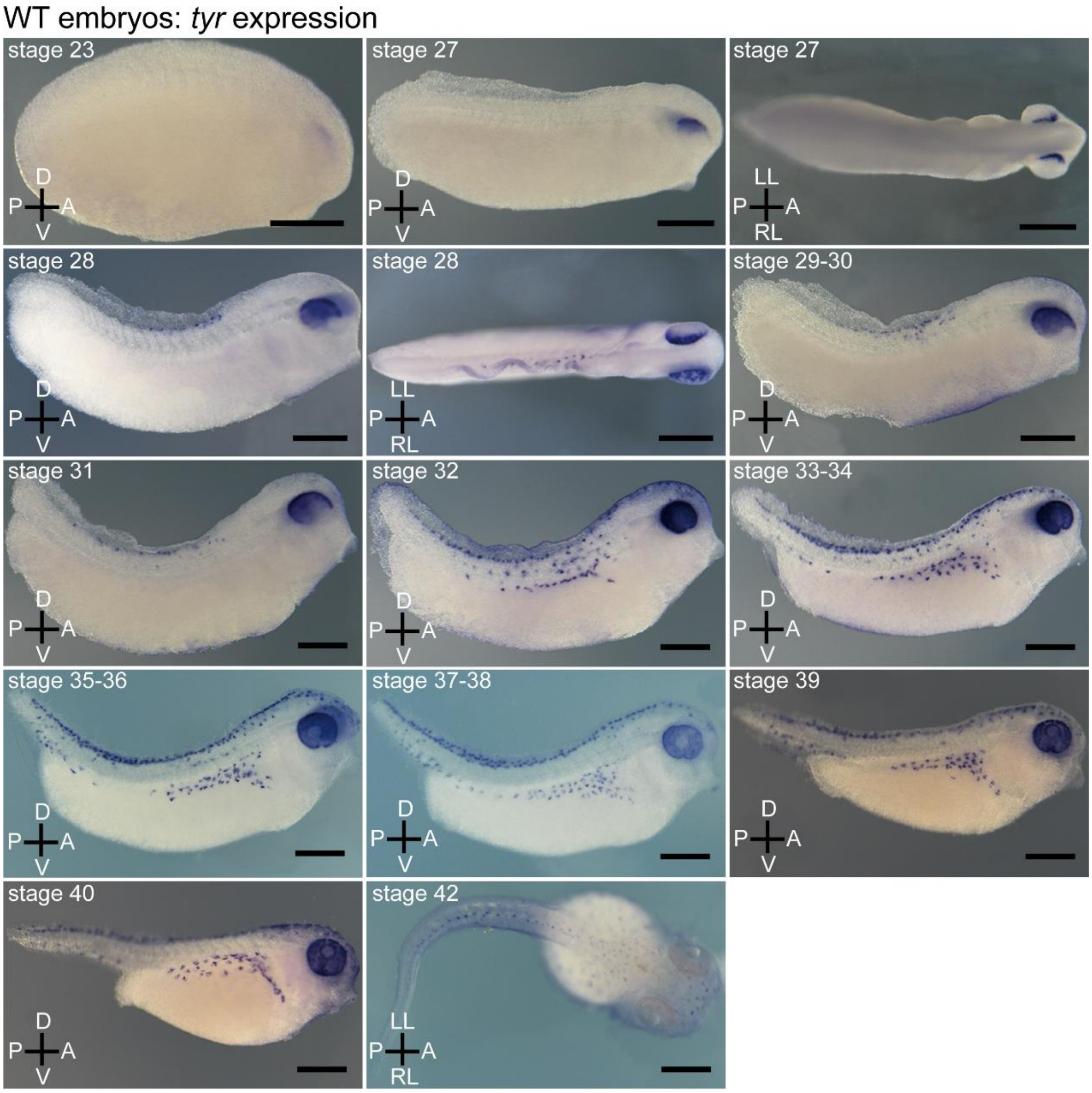

Supplementary Figure 13. The WISH results of *tyr* mRNA expression during embryonic development of WT *Xenopus tropicalis*. From stage 23 to stage 42, approximately 6-10 embryos per stage were used for in situ hybridization detection of *tyr* mRNA. **RL** represents right lateral view, **LL** represents left lateral view, **D** represents dorsal view, **V** represents ventral view, **P** represents posterior view, and **A** represents anterior view. Each scale bar is 0.5 mm.

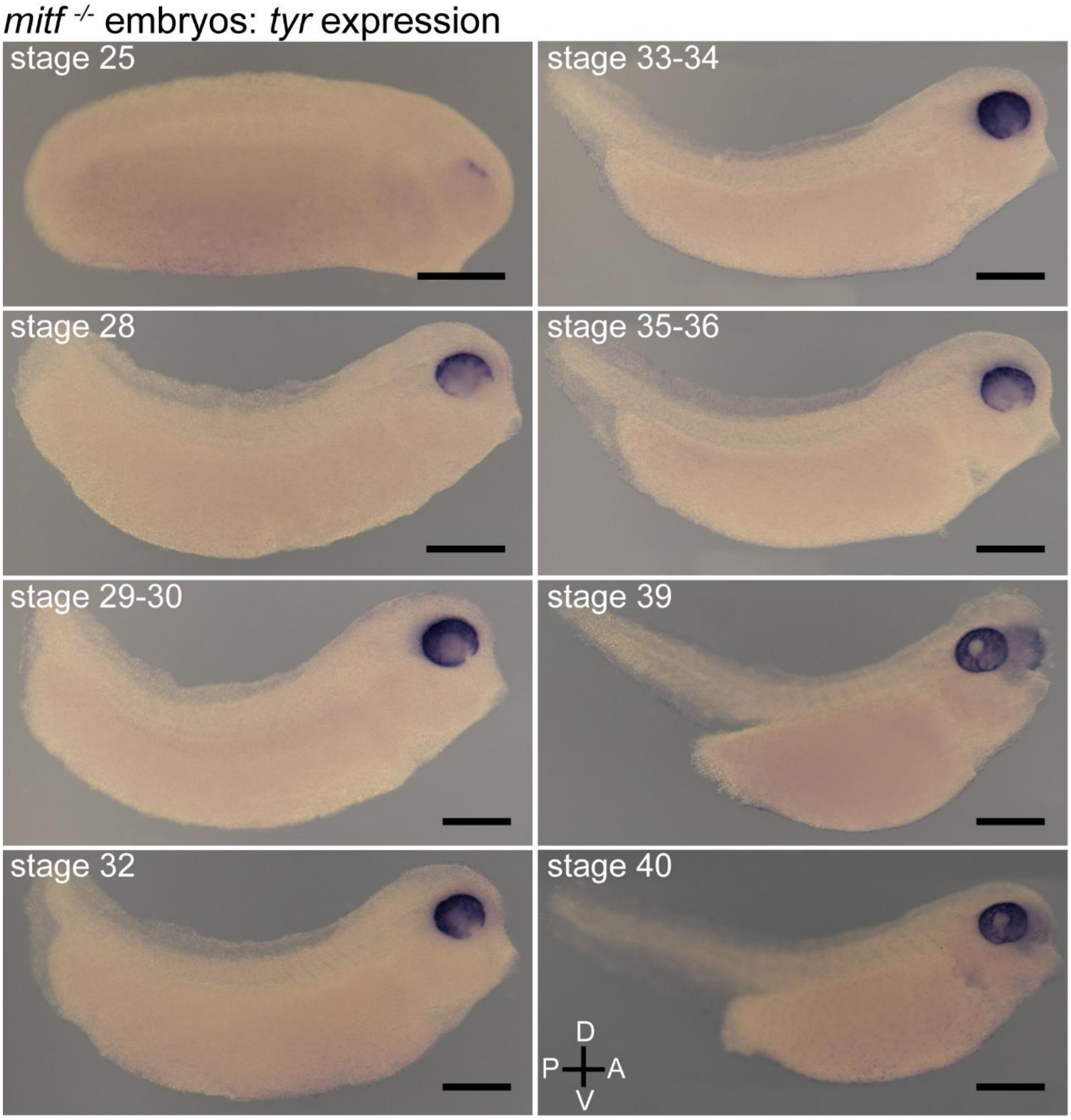

Supplementary Figure 14. The WISH results of *tyr* mRNA expression during embryonic development of *mitf*$^{-/-}$ *Xenopus tropicalis*. From stage 25 to stage 40, approximately 6-10 embryos per stage were used for in situ hybridization detection of *tyr* mRNA. **RL** represents right lateral view, **LL** represents left lateral view, **D** represents dorsal view, **V** represents ventral view, **P** represents posterior view, and **A** represents anterior view. Each scale bar is 0.5 mm.

WT embryos: *pmel* expression

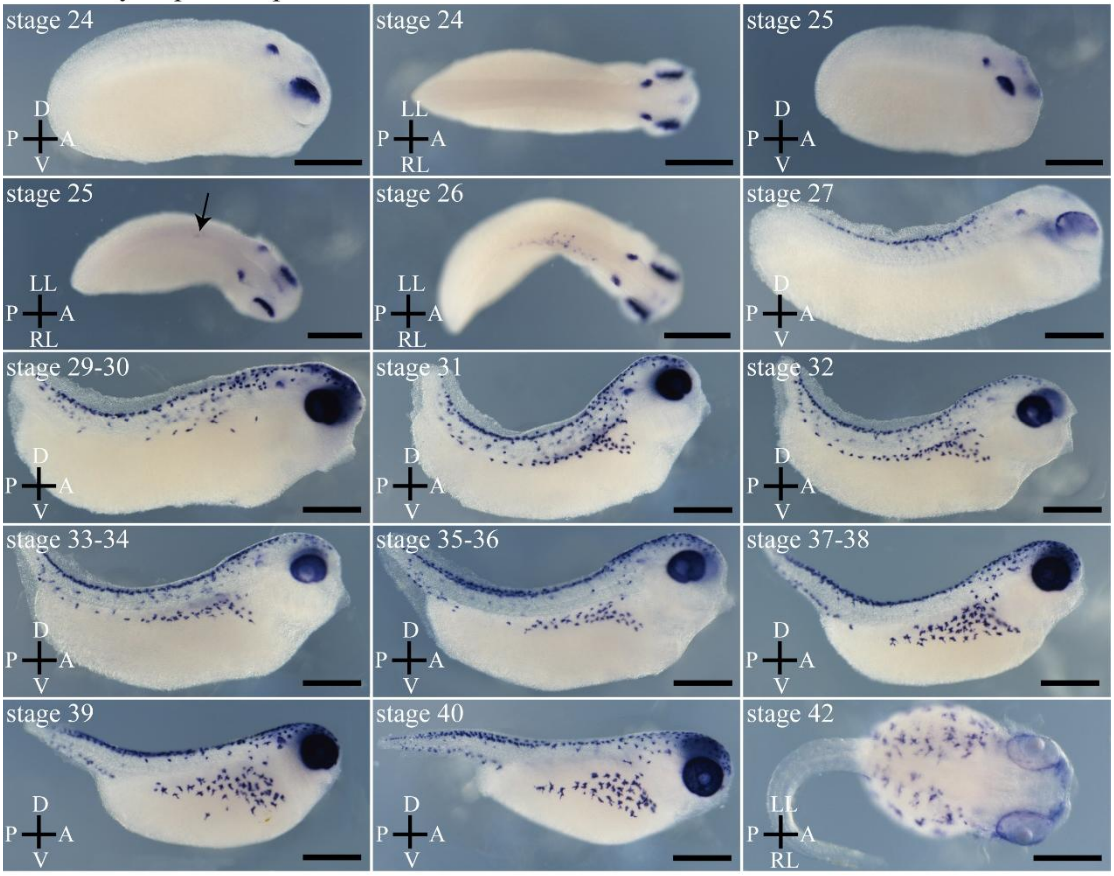

Supplementary Figure 15. The WISH results of *pmel* mRNA expression during embryonic development of WT *Xenopus tropicalis*. From stage 24 to stage 42, approximately 6-10 embryos per stage were used for in situ hybridization detection of *pmel* mRNA. **RL** represents right lateral view, **LL** represents left lateral view, **D** represents dorsal view, **V** represents ventral view, **P** represents posterior view, and **A** represents anterior view. Each scale bar is 0.5 mm.

mitf$^{-/-}$ embryos: *pmel* expression

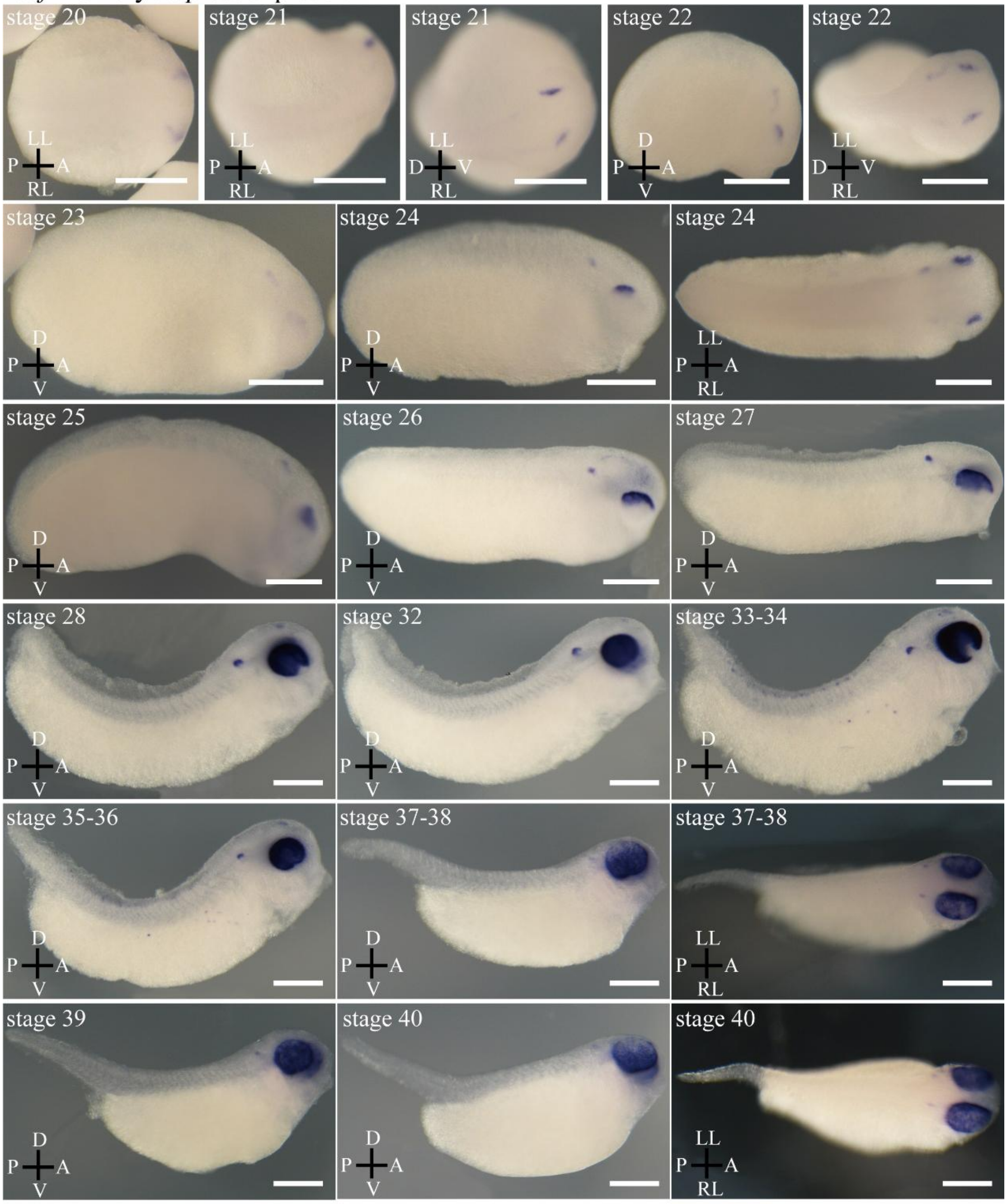

Supplementary Figure 16. The WISH results of *pmel* mRNA expression during embryonic development of *mitf*$^{-/-}$ *Xenopus tropicalis*. From stage 20 to stage 40, approximately 6-10 embryos per stage were used for in situ hybridization detection of *pmel* mRNA. **RL** represents right lateral view, **LL** represents left lateral view, **D** represents dorsal view, **V** represents ventral view, **P** represents posterior view, and **A** represents anterior view. Each scale bar is 0.5 mm.

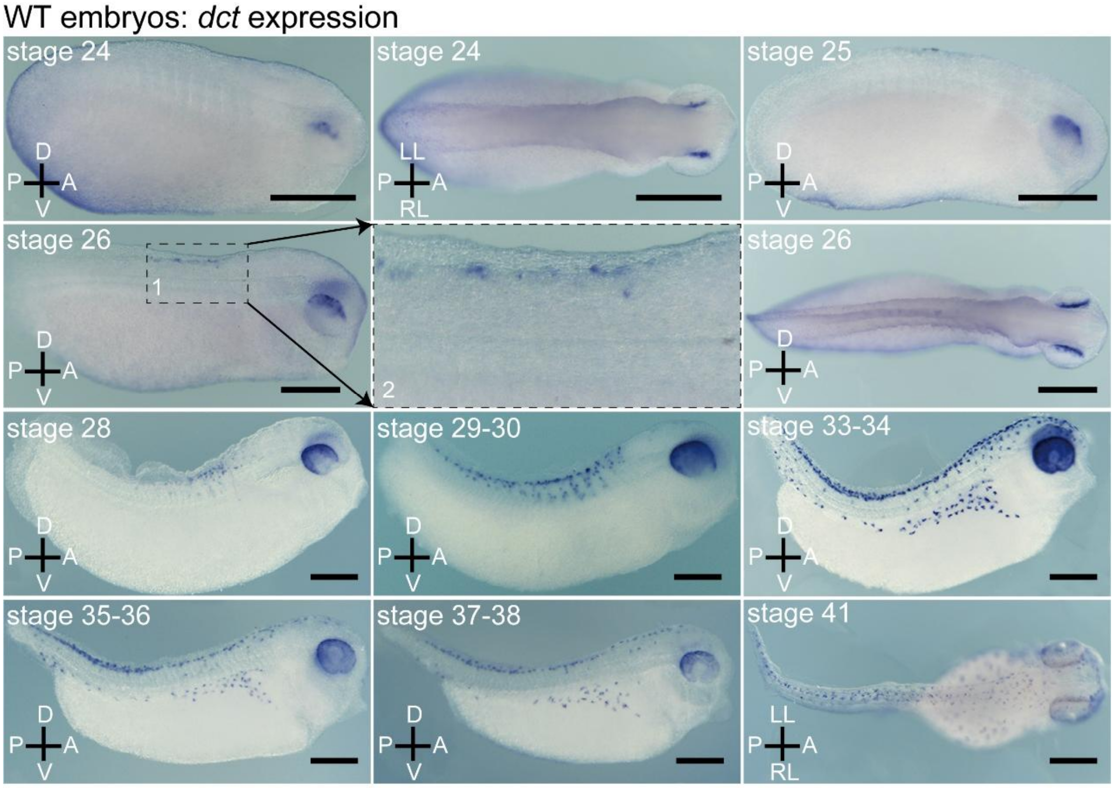

Supplementary Figure 17. The WISH results of *dct* mRNA expression during embryonic development of WT *Xenopus tropicalis*. From stage 24 to stage 41, approximately 6-10 embryos per stage were used for in situ hybridization detection of *dct* mRNA. **RL** represents right lateral view, **LL** represents left lateral view, **D** represents dorsal view, **V** represents ventral view, **P** represents posterior view, and **A** represents anterior view. Black dashed boxes **1** corresponded to magnified views of black dashed boxes **2**. Each scale bar is 0.5 mm.

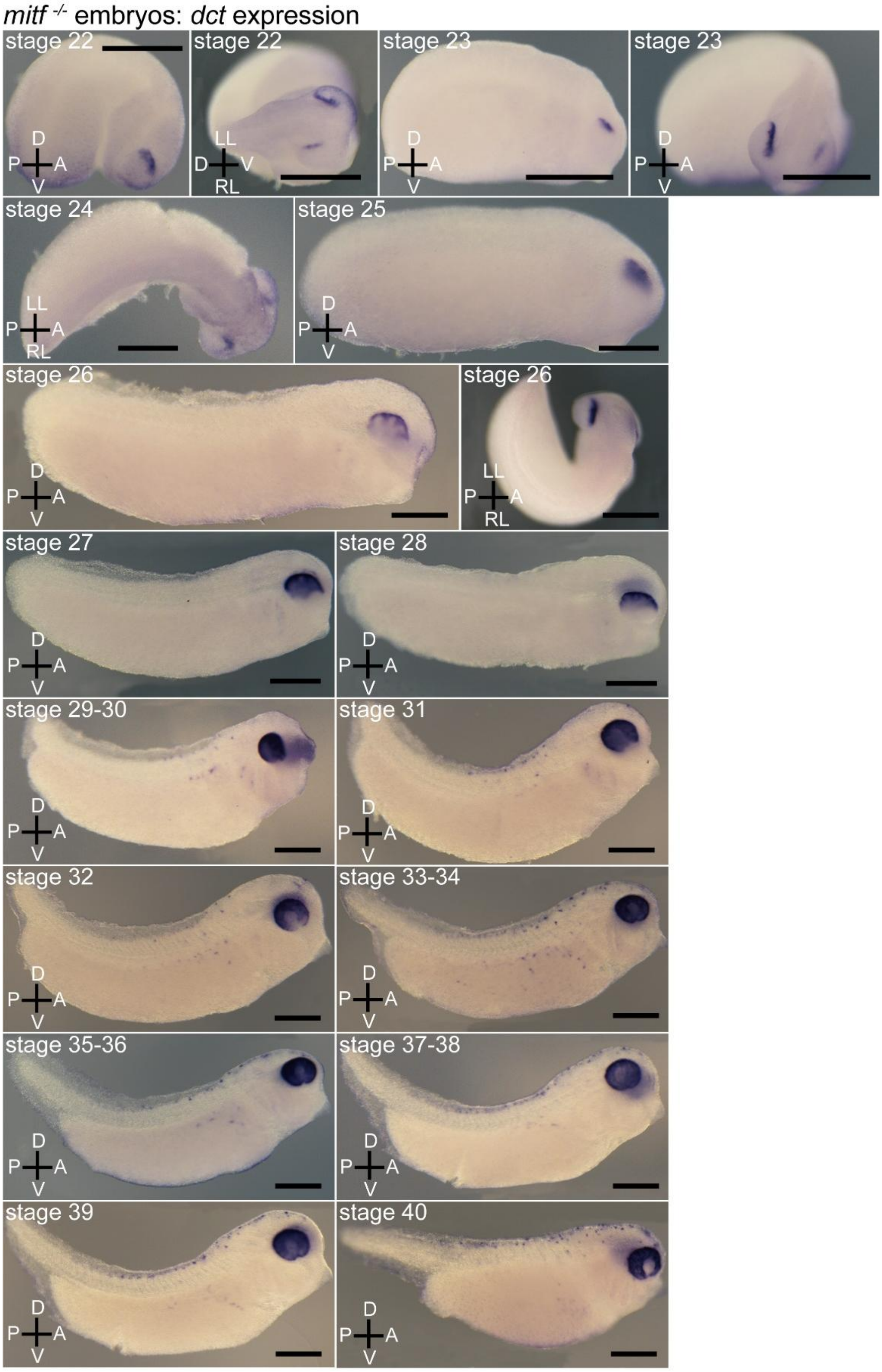

Supplementary Figure 18. The WISH results of *dct* mRNA expression during embryonic development of *mitf*$^{-/-}$ *Xenopus tropicalis*. From stage 22 to stage 40, approximately 6-10 embryos per stage were used for in situ hybridization detection of *dct* mRNA. **RL** represents right lateral view, **LL** represents left lateral view, **D** represents dorsal view, **V** represents ventral view, **P** represents posterior view, and **A** represents anterior view. Each scale bar is 0.5 mm.

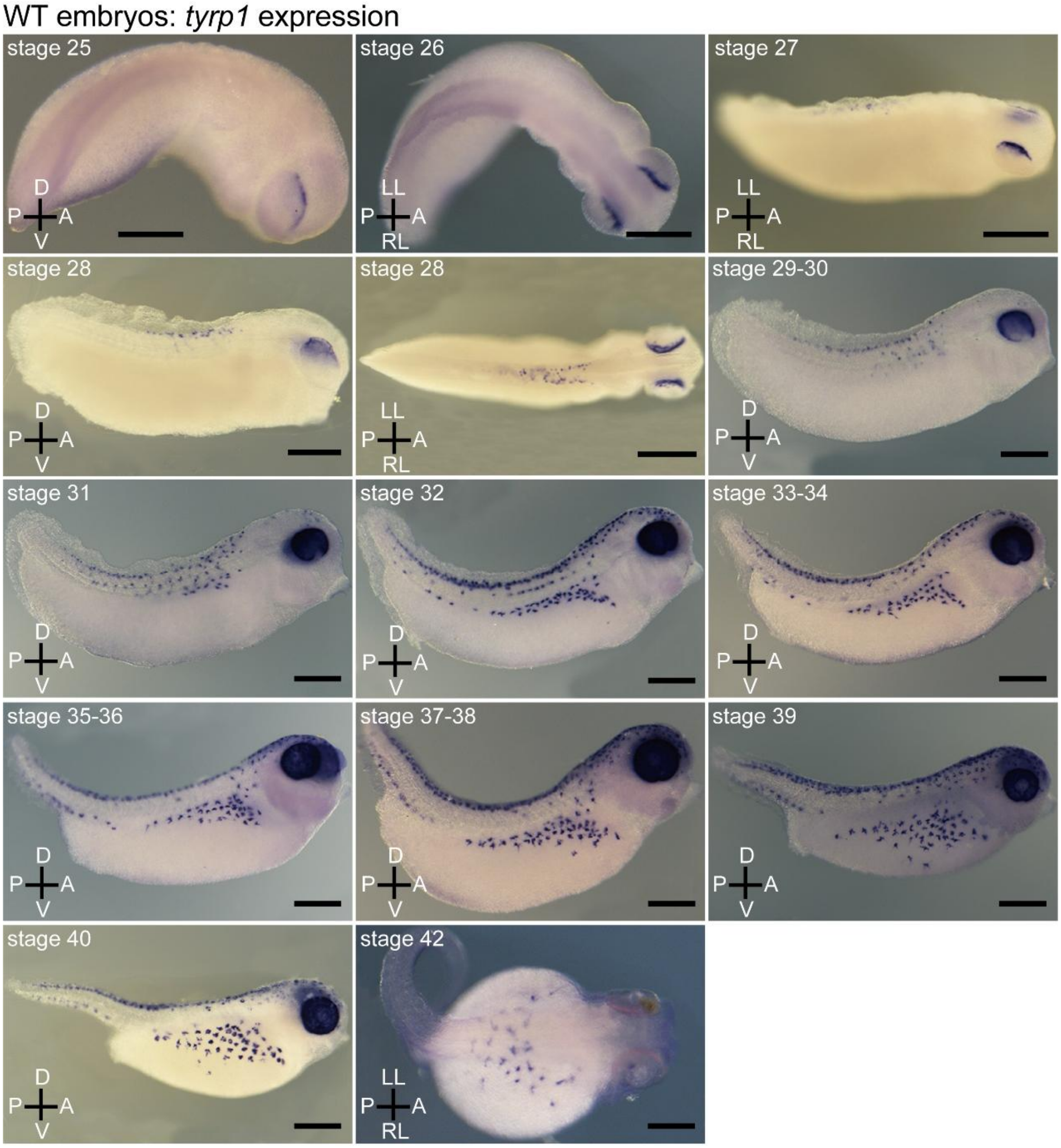

Supplementary Figure 19. The WISH results of *tyrp1* mRNA expression during embryonic development of WT *Xenopus tropicalis*. From stage 25 to stage 42, approximately 6-10 embryos per stage were used for in situ hybridization detection of *tyrp1* mRNA. **RL** represents right lateral view, **LL** represents left lateral view, **D** represents dorsal view, **V** represents ventral view, **P** represents posterior view, and **A** represents anterior view. Each scale bar is 0.5 mm.

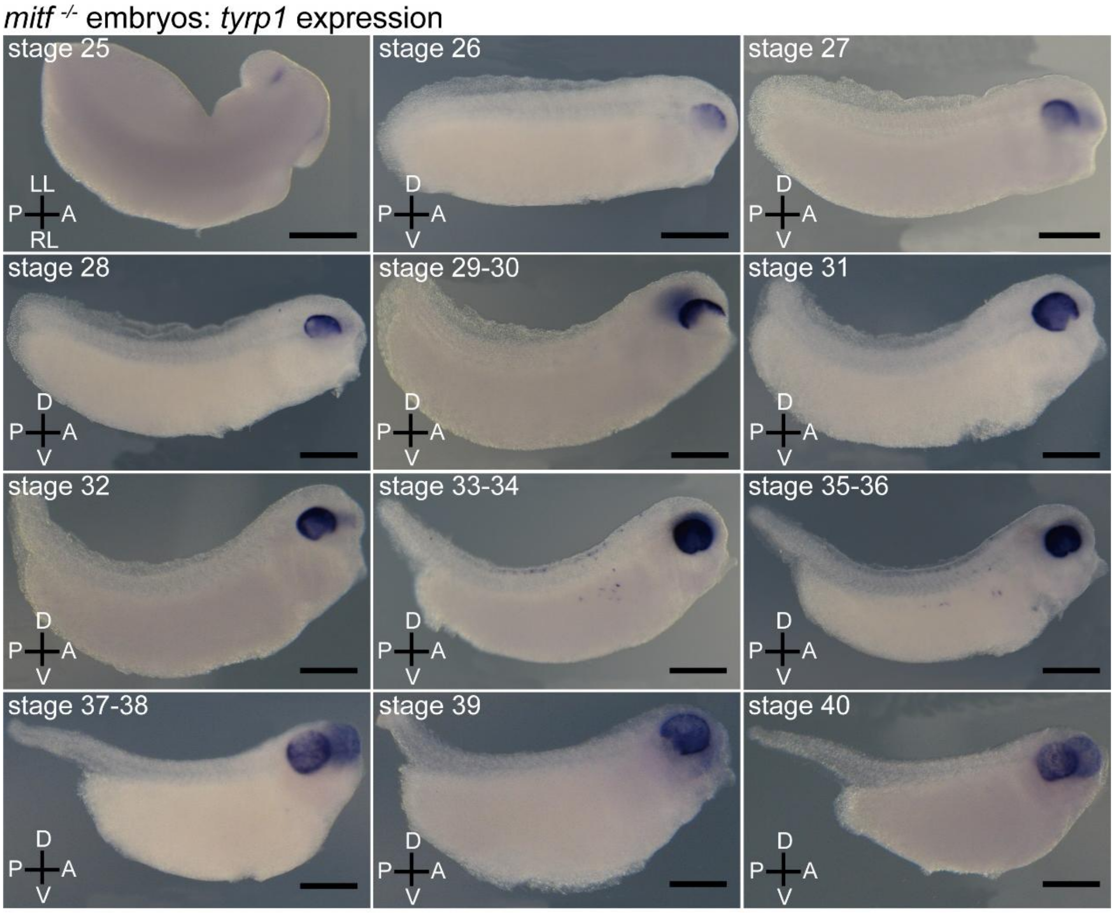

Supplementary Figure 20. The WISH results of *tyrp1* mRNA expression during embryonic development of *mitf$^{-/-}$* *Xenopus tropicalis*. From stage 25 to stage 40, approximately 6-10 embryos per stage were used for in situ hybridization detection of *tyrp1* mRNA. **RL** represents right lateral view, **LL** represents left lateral view, **D** represents dorsal view, **V** represents ventral view, **P** represents posterior view, and **A** represents anterior view. Each scale bar is 0.5 mm.

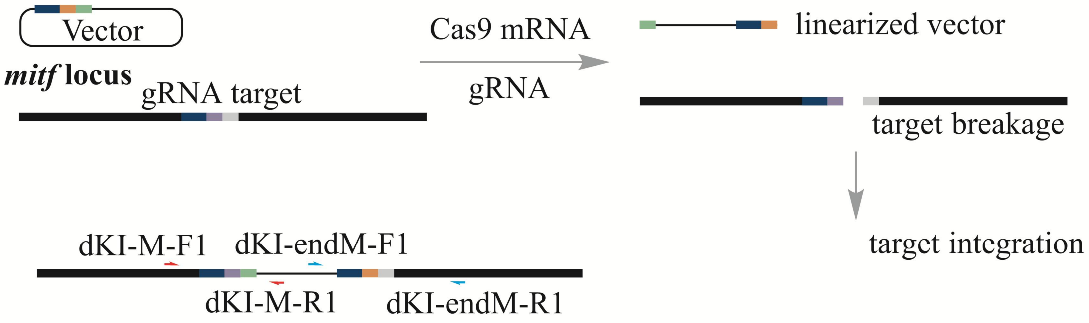

Supplementary Figure 21. Diagram of CRISPR/Cas9-mediated targeted integration of exogenous genes via non-homologous end joining repair, as well as genotyping detection after targeted integration. The primer sequences shown in the Figure were detailed in Supplementary Data 3.

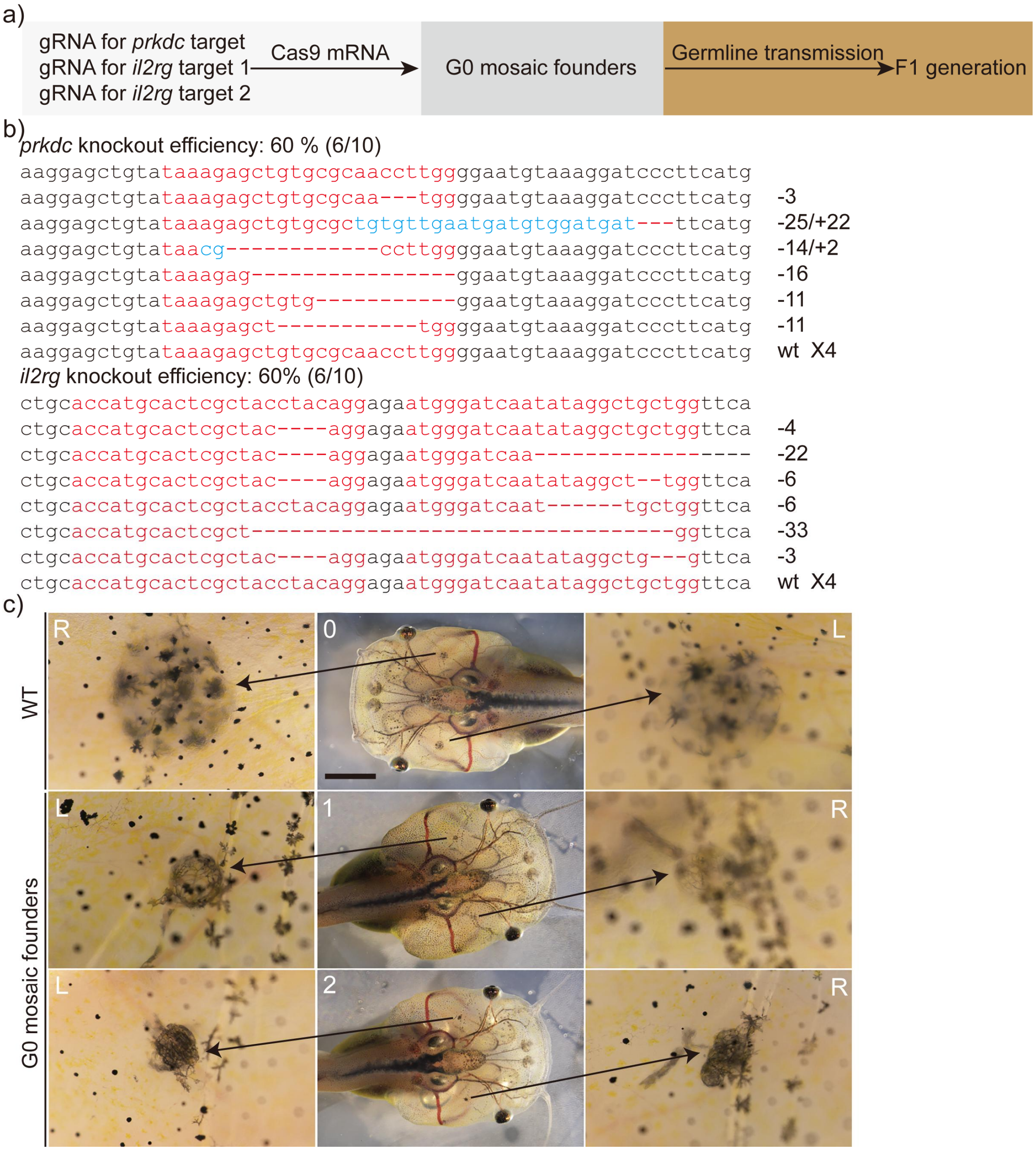

Supplementary Figure 22. Abnormal thymus development occurs in *prkdc/il2rg* double-knockout *Xenopus tropicalis*. **(a)** The strategy for establishing *prkdc/il2rg* double-knockout *Xenopus tropicalis* via CRISPR/Cas9 was depicted. **(b)** The results of Sanger sequencing indicating the knockout efficiency of *prkdc* and *il2rg* were presented. The gRNA target sequence was represented by red letters, the insertion sequence by blue letters, and base deletions by red dashed lines. **(c)** By using CRISPR/Cas9 to target both the *prkdc* and *il2rg* genes, some tadpoles in the founder generation (G0) at stage 56 displayed abnormal thymus development, indicated by the black arrows. 53% of randomly selected G0 tadpoles (72/135) at stage 55 exhibited abnormal thymus development. Representative images of G0 tadpoles showing the abnormal thymus development were displayed here. **R** represents the right thymus view, **L** represents the left thymus view, **0-2** refer to the WT tadpole and two mosaic tadpoles, respectively. The scale bar is 1 mm.

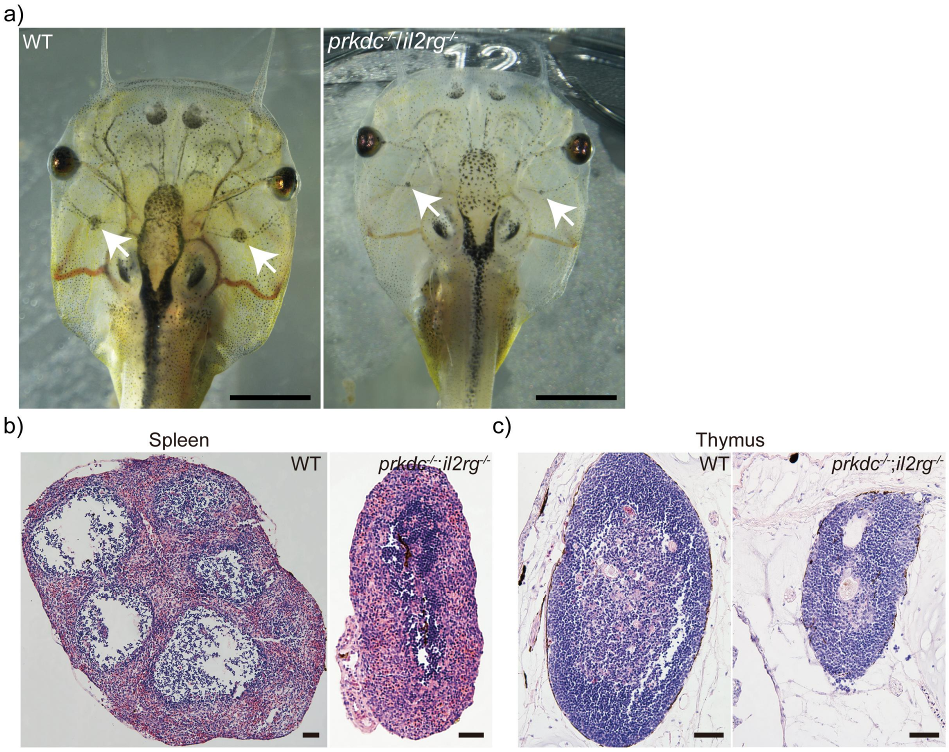

Supplementary Figure 23. (a) The thymuses indicated by white arrows of WT and *prkdc*$^{-/-}$/*il2rg*$^{-/-}$ tadpoles at stage 55 were compared. Six WT and six *prkdc*$^{-/-}$/*il2rg*$^{-/-}$ tadpoles were observed, and the representative results were presented here. **(b)-(c)** The representative results of histological examination were shown. H&E staining was used in spleen (**b**) and thymus (**c**) of eight WT and F1 *prkdc*$^{-/-}$/*il2rg*$^{-/-}$ tadpoles at stage 57. The scale bar in **(a)**, 1 mm; Scale bar in **(b)** and **(c)**, 50 µm.

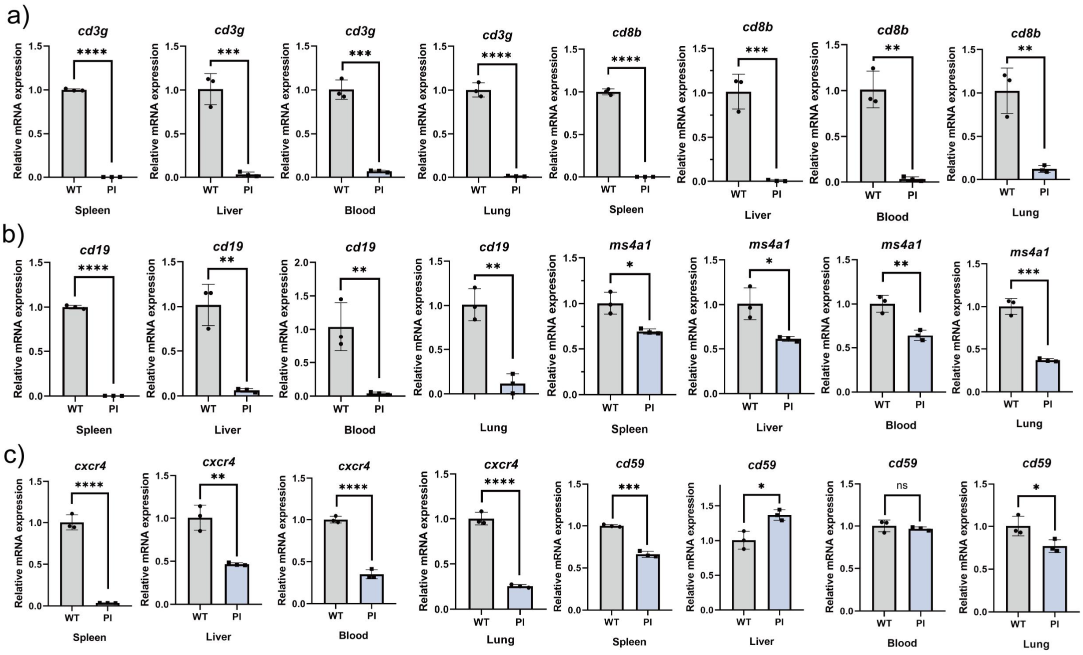

Supplementary Figure 24. The expression of T-cell (a), B-cell (b), and NK-cell (c) marker genes in *prkdc*$^{-/-}$/*il2rg*$^{-/-}$ (PI) *Xenopus tropicalis* is shown. Graphpad prism 9 was used for the t-test (*P < 0.05, **P < 0.01,***P < 0.001, ****P < 0.0001).

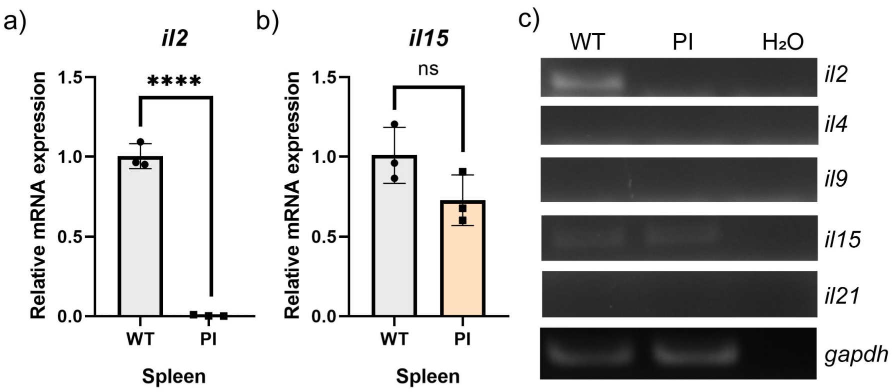

Supplementary Figure 25. The expression of five cytokines (Il-2, Il-4, Il-9, Il-15, and Il-21) in *prkdc*$^{-/-}$*/il2rg*$^{-/-}$*mitf*$^{-/-}$ (PIM) *Xenopus tropicalis* is shown. *gapdh* was used as a RNA loading control. The original data for the RT-PCR in (c) can be found in Supplementary Fig. 34. Graphpad prism 9 was used for the t-test (****P < 0.0001).

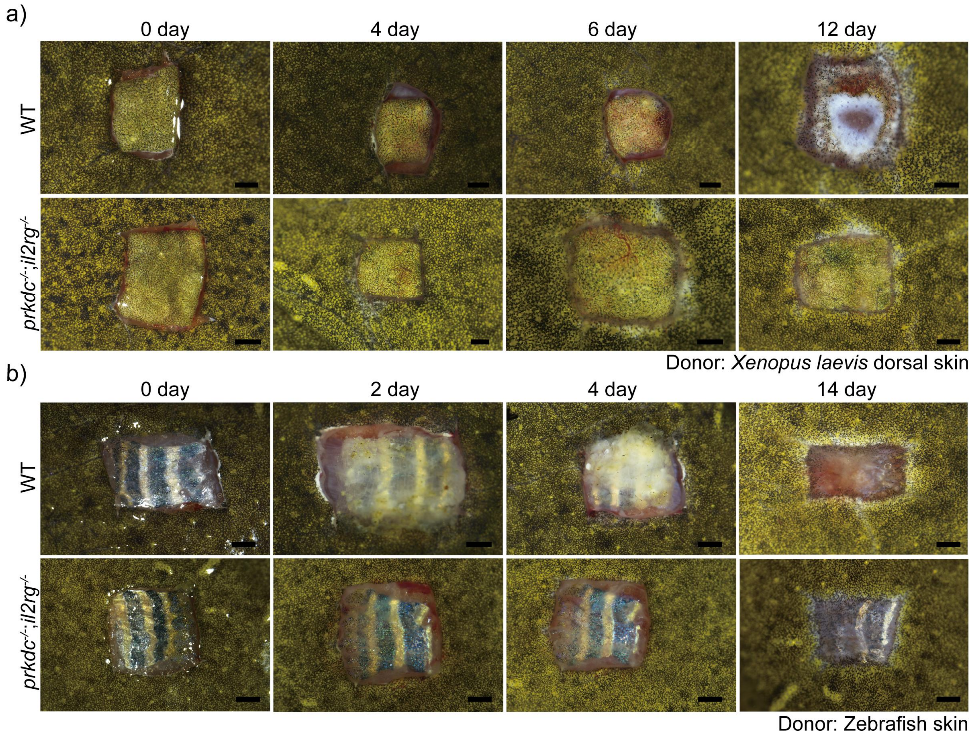

Supplementary Figure 26. Results of skin xenotransplantation in WT and *prkdc*$^{-/-}$*/il2rg*$^{-/-}$ *Xenopus tropicalis*. (a) Six adult frogs, consisting of three WT and three *prkdc*$^{-/-}$*/il2rg*$^{-/-}$ *Xenopus tropicalis*, aged 6 months were selected as recipients for skin transplantation. The skin grafts were obtained from three *Xenopus laevis* donors aged 2 years. Specifically, the dorsal skins of the donors (approximately 5 mm x 5 mm in size) were used to graft onto the dorsal skin of the recipients. The grafting outcomes were monitored for a 12-day period post-transplantation. The representative results were presented here. **(b)** Six adult *Xenopus tropicalis* frogs, consisting of three WT and three *prkdc*$^{-/-}$*/il2rg*$^{-/-}$, aged 6 months were employed as recipients for skin graft transplantation. The donors were three 2-month-old zebrafish whose lateral skins (approximately 5 mm x 5 mm in size) were grafted onto the dorsal skin of the recipients. The grafting outcomes were observed over a 14-day period post-transplantation.The representative results were presented here. Scale bar is 1 mm.

mitf
allele 1
agcctc----gattacattcgcaaact -4bp
allele 2
agcct-------ttacattcgcaaact -7bp

prkdc
allele 1
tataaagagctgtg-----------gg -11
allele 2
tataaagagct-----------tggg g -11

il2rg
allele 1
ctgcaccatgcactcgctac----aggagaatgggatcaatataggctgctggttca -4
allele 2
ctgcaccatgcactcgctac----aggagaatgggatcaa----------------- -22

Supplementary Figure 27. The genotype of *mitf*$^{-/-}$/*prkdc*$^{-/-}$/*il2rg*$^{-/-}$ *Xenopus tropicalis* was shown.

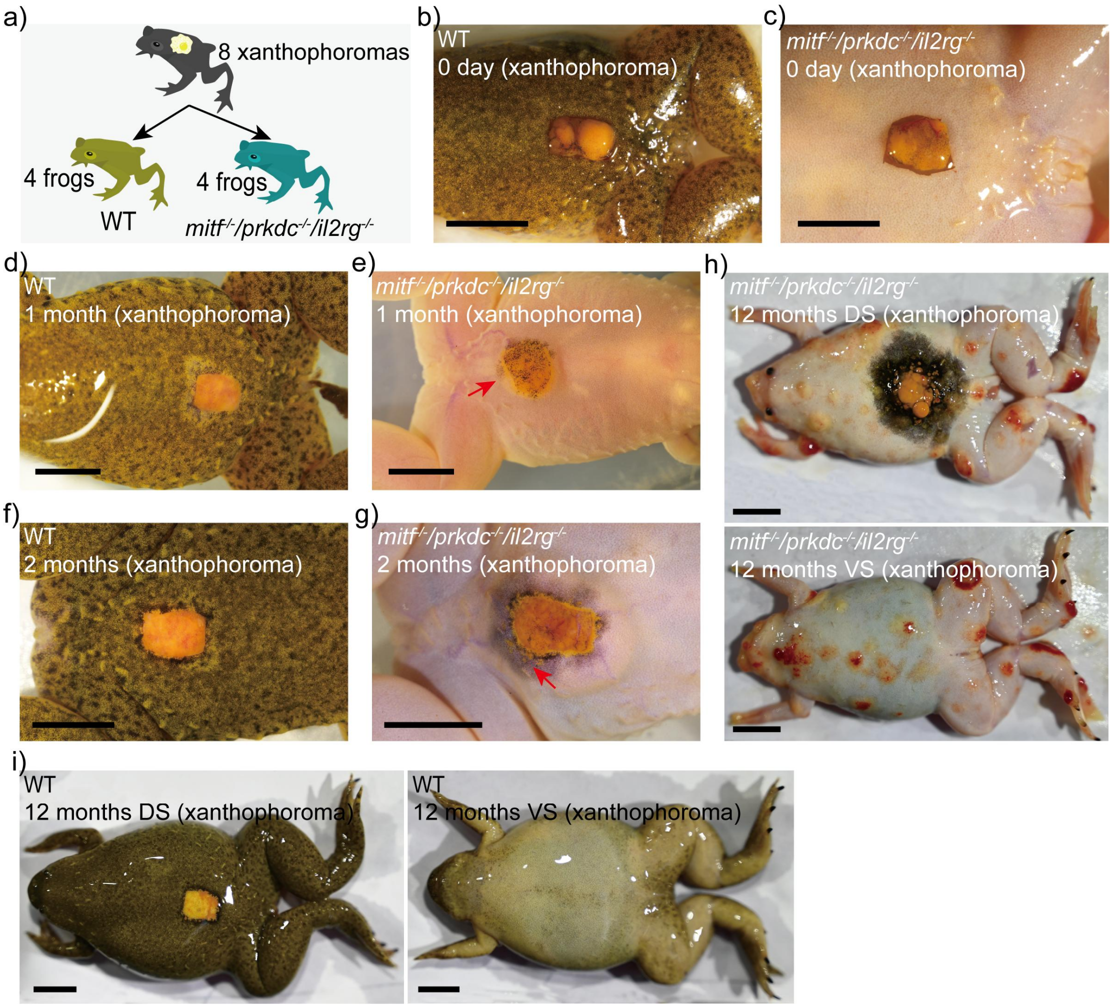

Supplementary Figure 28. Results of allogeneic transplantations of xanthophoromas in *mitf*$^{-/-}$/*prkdc*$^{-/-}$/*il2rg*$^{-/-}$ *Xenopus tropicalis*. (a) Eight frogs, consisting of four WT and four *mitf*$^{-/-}$/*prkdc*$^{-/-}$/*il2rg*$^{-/-}$ *Xenopus tropicalis*, aged 3 months were employed as recipients for xanthophoroma transplantations. The donors were eight xanthophoromas (approximately 5 mm x 5 mm in size) from eight *cdkn2b*$^{-/-}$;*mitf*-BRAF^{V600E} tadpoles at stage 58, which spontaneously formed xanthophoromas. The donor xanthophoromas were grafted onto the dorsal skin of the recipients. **(b)-(i)** The grafting outcomes were observed over a 1-year period post-transplantation. The representative results were presented here. Scale bar is 1 cm.

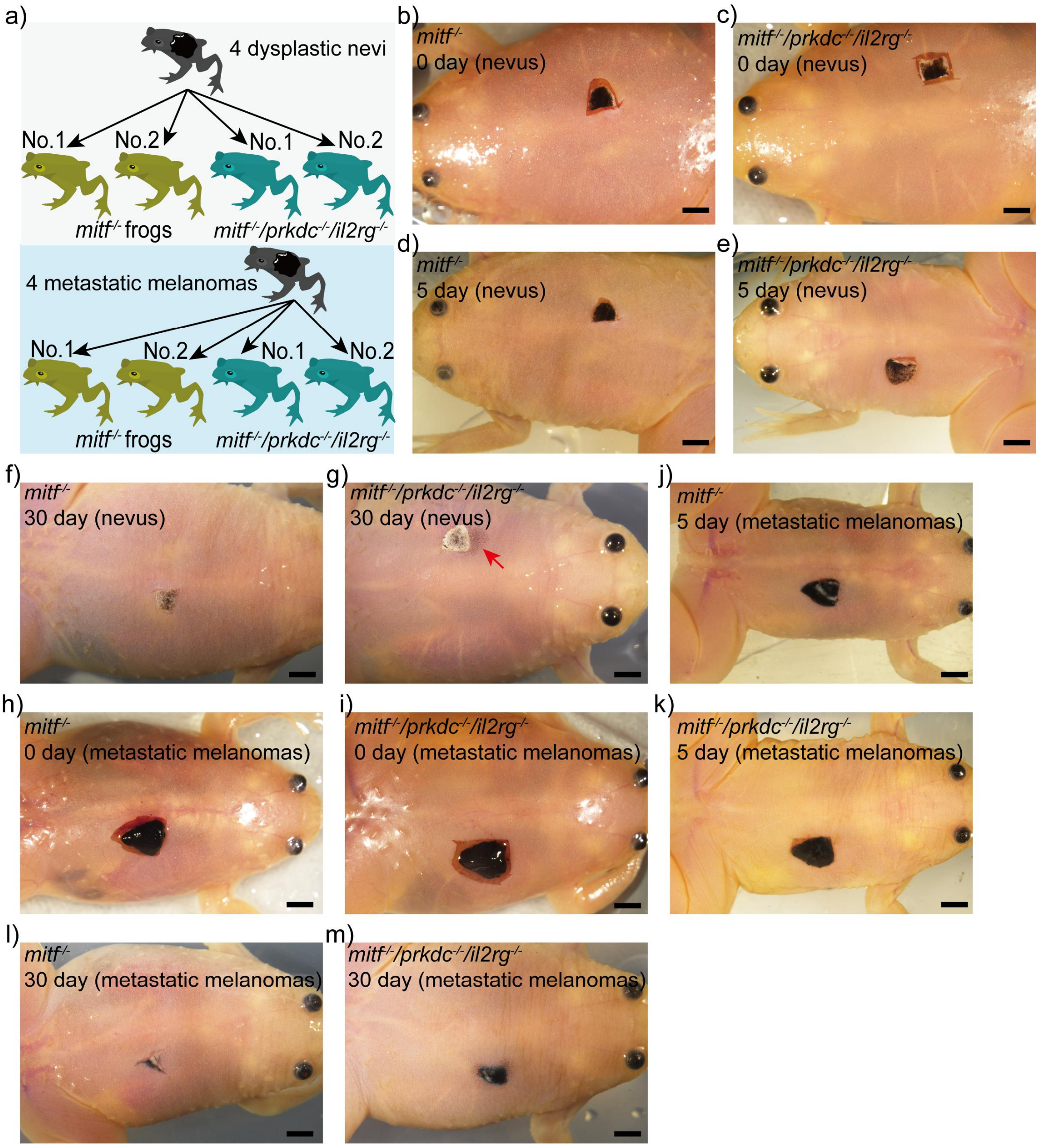

Supplementary Figure 29. Results of allogeneic transplantations of melanomas in *mitf$^{-/-}$/prkdc$^{-/-}$/il2rg$^{-/-}$ Xenopus tropicalis*. (a) Eight frogs, consisting of four *mitf$^{-/-}$* and four *mitf$^{-/-}$/prkdc$^{-/-}$/il2rg$^{-/-}$ Xenopus tropicalis*, aged 3 months were employed as recipients for melanoma transplantations. The donors were four dysplastic nevi and four metastatic melanomas (approximately 2 mm x 2 mm in size) from two 1.5-year-old *tp53$^{-/-}$ Xenopus tropicalis*, which spontaneously formed melanomas. The donor nevi and melanomas were grafted onto the dorsal skin of the recipients as shown in the diagram. **(b)-(m)** The grafting outcomes were observed over a 30-day period post-transplantation. The representative results were presented here. Scale bar is 1 mm.

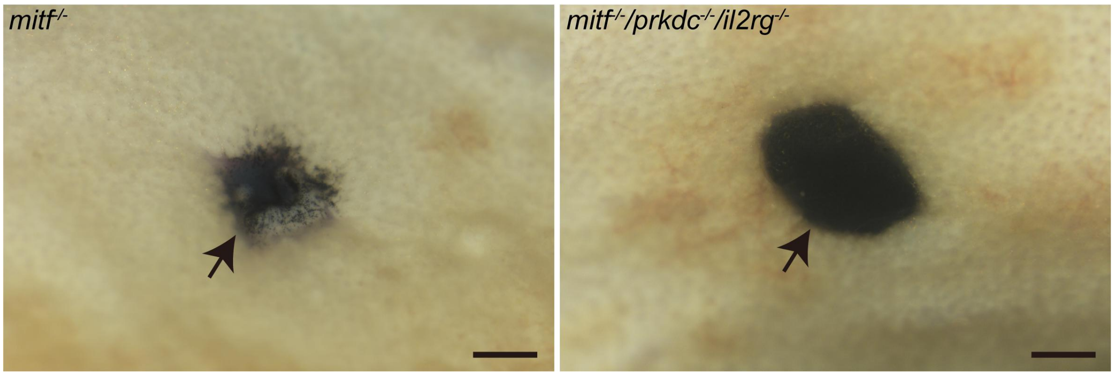

Supplementary Figure 30. The findings from the transplantation of dysplastic nevi in *mitf*$^{-/-}$ and *mitf*$^{-/-}$*/prkdc*$^{-/-}$*/il2rg*$^{-/-}$ *Xenopus tropicalis* were presented. Donor dysplastic nevi were obtained from 14-month-old *cdkn2b*$^{-/-}$*/tp53*$^{-/-}$ *Xenopus tropicalis*, which spontaneously developed nevi. Two distinct dysplastic nevi samples were grafted onto the dorsal skin of a one-year-old *mitf*$^{-/-}$ and an *mitf*$^{-/-}$*/prkdc*$^{-/-}$*/il2rg*$^{-/-}$ *Xenopus tropicalis* individually. The accompanying images depict outcomes observed on the 40th day post-transplantation, with black arrows highlighting the transplanted dysplastic nevi. The scale bar is 1 mm.

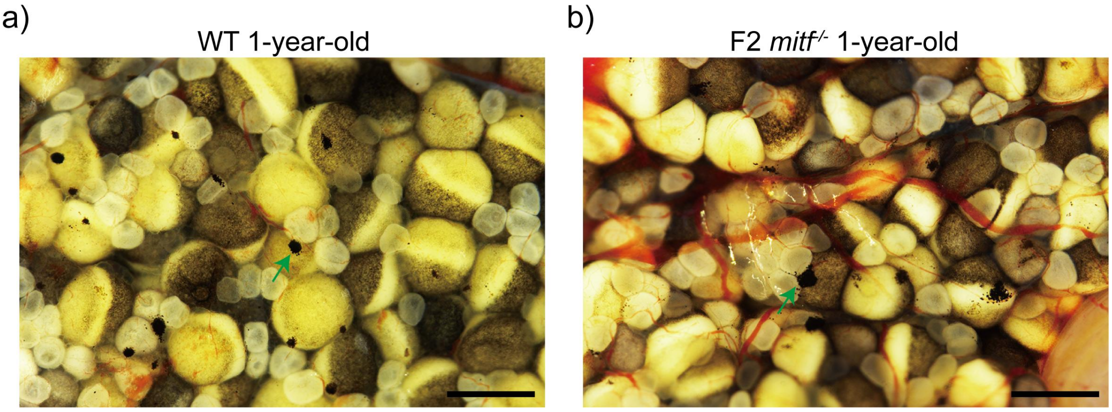

Supplementary Figure 31. Pigment deposition in oocytes of one-year-old WT and *mitf*$^{-/-}$ *Xenopus tropicalis*. Pigment deposition in the oocyte of one-year-old WT (**a**) and *mitf*$^{-/-}$ (**b**) *Xenopus tropicalis* was displayed. Melanomacrophage centers are indicated by green arrows. Scale bar is 1 mm.

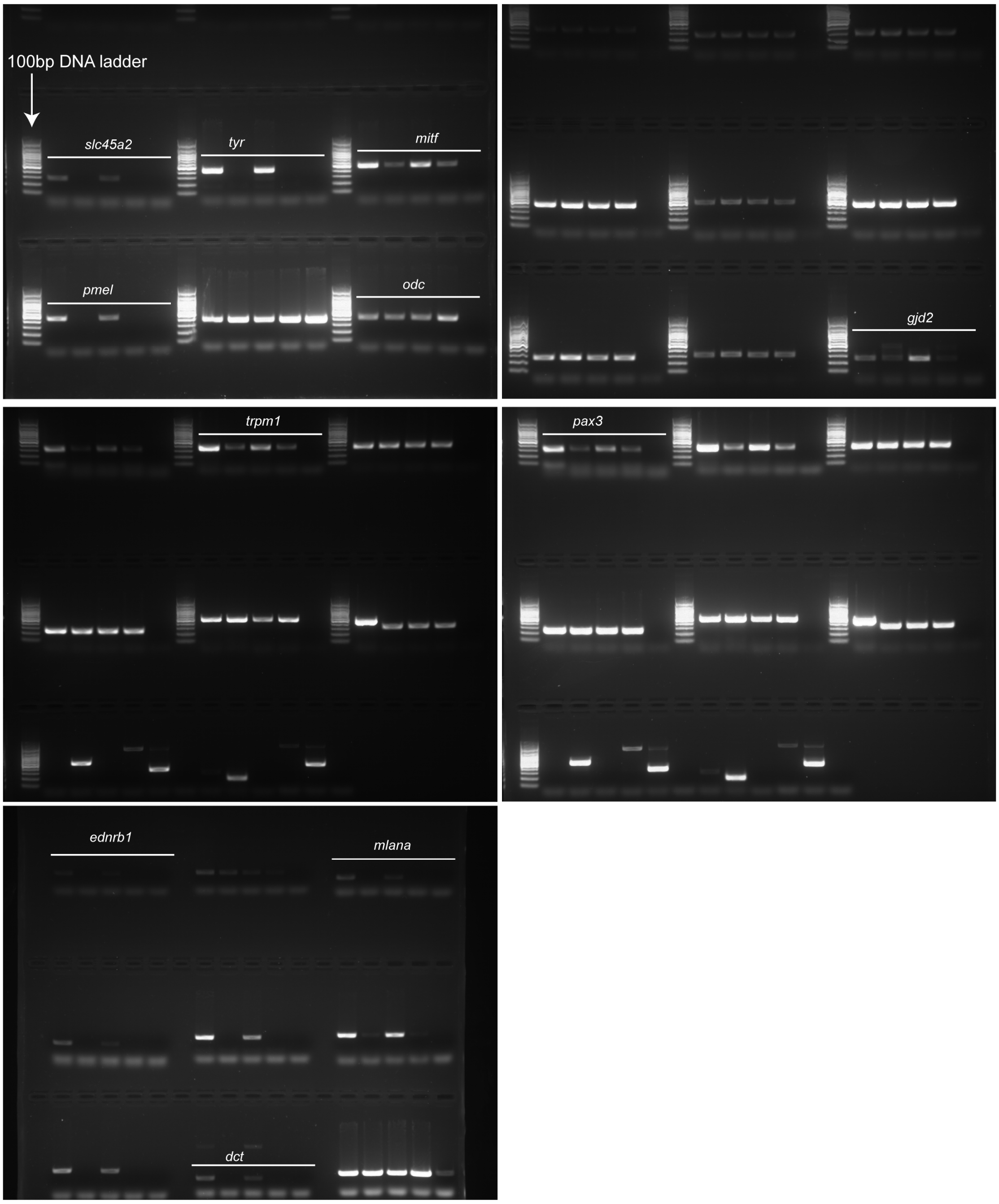

Supplementary Figure 32. Original RT-PCR data for Figure 4.b.

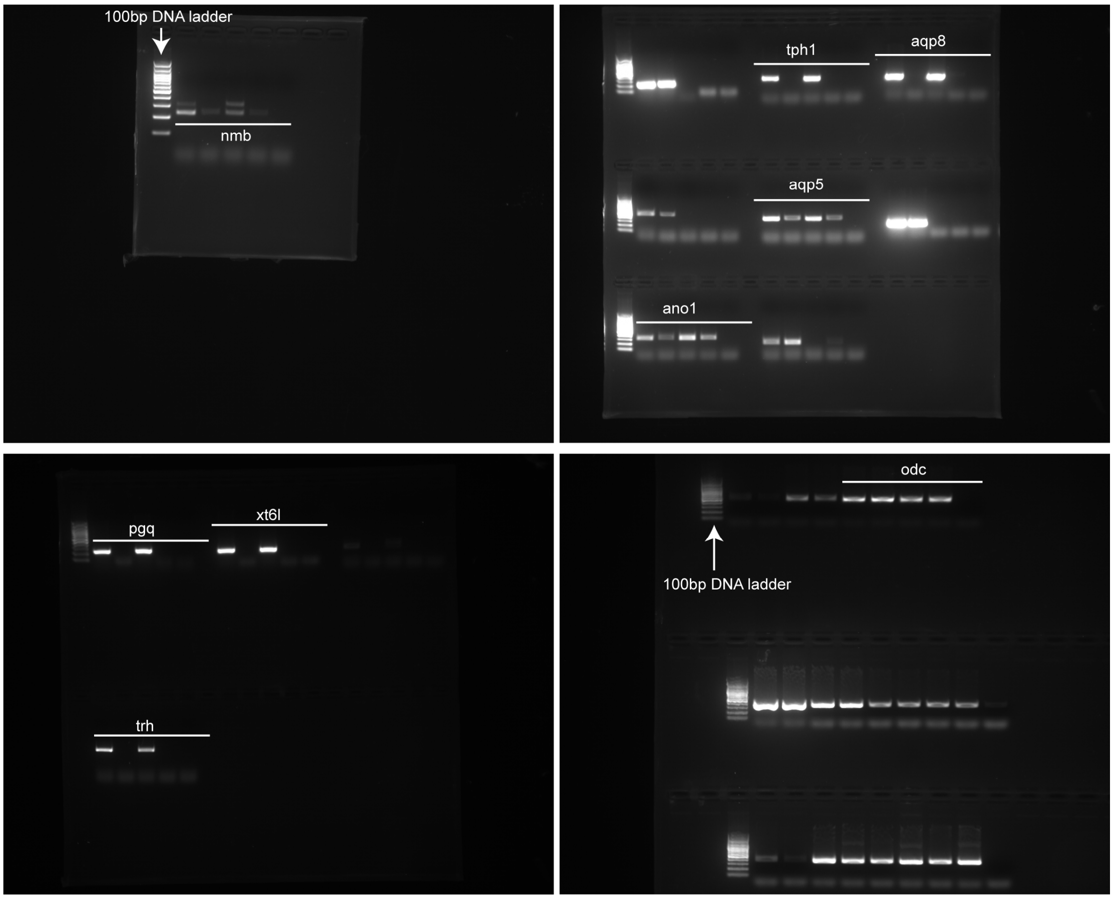

Supplementary Figure 33. Original RT-PCR data for Figure 4.c-e.

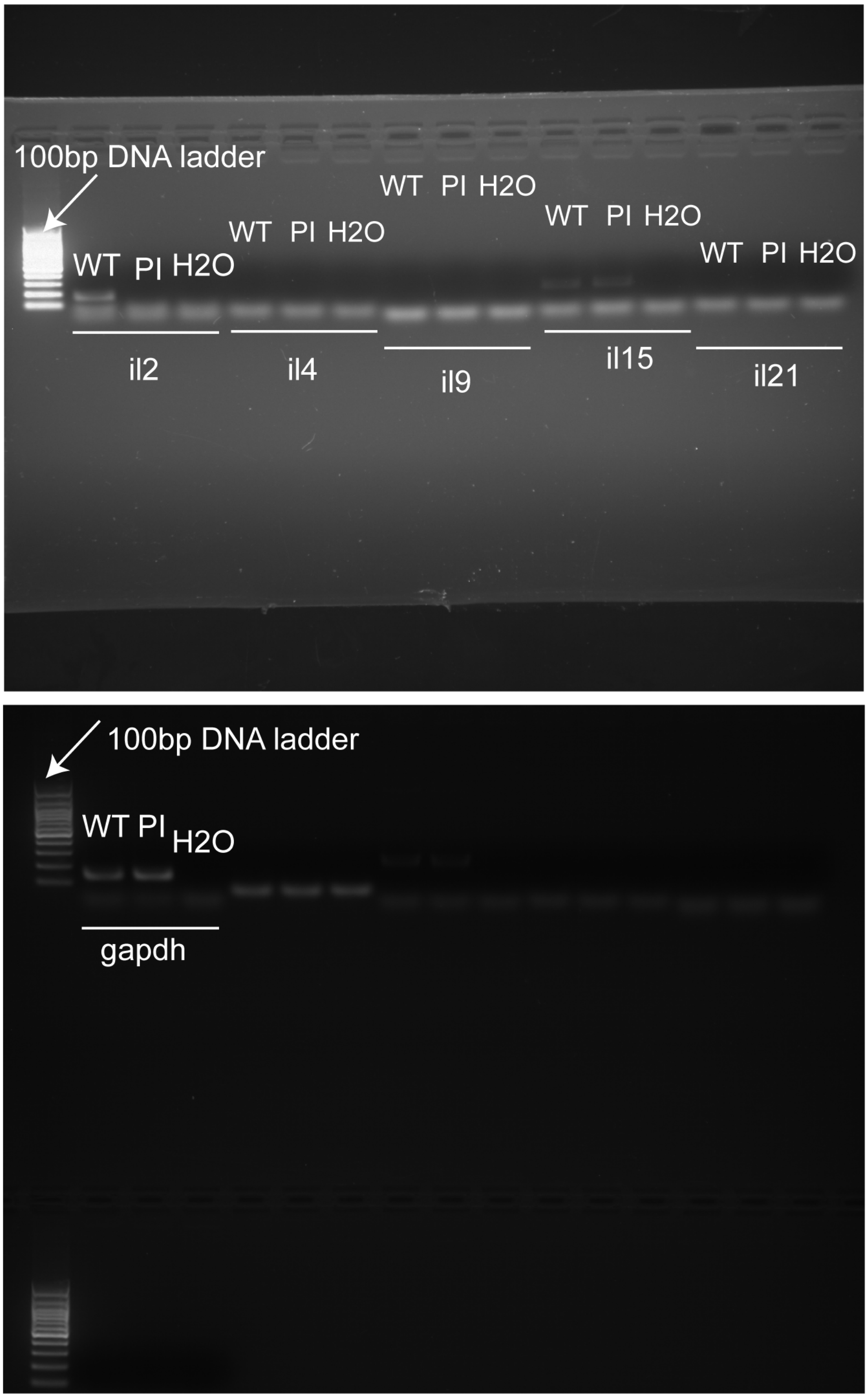

Supplementary Figure 34. Original RT-PCR data for Supplementary Figure 25.c.
