# Supplementary material for: Revealing mitf functions and visualizing allografted tumor metastasis in colorless and immunodeficient Xenopus tropicalis
Source: Commun Biol. 2024 Mar 5;7:275. doi: 10.1038/s42003-024-05967-3 (PMC10915148; doi:10.1038/s42003-024-05967-3)
Supplement: Supplementary file 3 — Description of additional supplementary files [file 42003_2024_5967_MOESM3_ESM.docx]

Description of Additional Supplementary Files

**File name:** Supplementary Data 1

**Description:** Genome-wide potential off-target sites of the gRNA-T7 were predicted by the CRISPOR.

**File name:** Supplementary Data 2

**Description:** Target information and primer sequences for off-target detection.

**File name:** Supplementary Data 3

**Description:** The primers (from 5' to 3' end) used in this project.

**File name:** Supplementary Data 4

**Description:** Vectors for genes targeted integration.
