# Supplementary material for: Revealing mitf functions and visualizing allografted tumor metastasis in colorless and immunodeficient Xenopus tropicalis
Source: Commun Biol. 2024 Mar 5;7:275. doi: 10.1038/s42003-024-05967-3 (PMC10915148; doi:10.1038/s42003-024-05967-3)
Supplement: Supplementary file 8 — Reporting Summary [file 42003_2024_5967_MOESM8_ESM.pdf]

nature portfolio

Corresponding author(s): Rensen Ran

Last updated by author(s): Jan 23, 2024

Reporting Summary

Nature Portfolio wishes to improve the reproducibility of the work that we publish. This form provides structure for consistency and transparency in reporting. For further information on Nature Portfolio policies, see our Editorial Policies and the Editorial Policy Checklist.

Statistics

For all statistical analyses, confirm that the following items are present in the figure legend, table legend, main text, or Methods section.

n/a	Confirmed	
☐	☒	The exact sample size (*n*) for each experimental group/condition, given as a discrete number and unit of measurement
☐	☒	A statement on whether measurements were taken from distinct samples or whether the same sample was measured repeatedly
☐	☒	The statistical test(s) used AND whether they are one- or two-sided<br>*Only common tests should be described solely by name; describe more complex techniques in the Methods section.*
☒	☐	A description of all covariates tested
☒	☐	A description of any assumptions or corrections, such as tests of normality and adjustment for multiple comparisons
☒	☐	A full description of the statistical parameters including central tendency (e.g. means) or other basic estimates (e.g. regression coefficient) AND variation (e.g. standard deviation) or associated estimates of uncertainty (e.g. confidence intervals)
☒	☐	For null hypothesis testing, the test statistic (e.g. *F*, *t*, *r*) with confidence intervals, effect sizes, degrees of freedom and *P* value noted<br>*Give P values as exact values whenever suitable.*
☒	☐	For Bayesian analysis, information on the choice of priors and Markov chain Monte Carlo settings
☒	☐	For hierarchical and complex designs, identification of the appropriate level for tests and full reporting of outcomes
☒	☐	Estimates of effect sizes (e.g. Cohen's *d*, Pearson's *r*), indicating how they were calculated

Our web collection on statistics for biologists contains articles on many of the points above.

Software and code

Policy information about availability of computer code

Data collection	N/A
Data analysis	The web tool CRISPOR was used for predicting off-target sites of CRISPR-Cas9 knockout. The web tool Clustal Omega was used in aligning protein sequence crossing species. The Graphpad Prism9 was used for statistical analysis.

For manuscripts utilizing custom algorithms or software that are central to the research but not yet described in published literature, software must be made available to editors and reviewers. We strongly encourage code deposition in a community repository (e.g. GitHub). See the Nature Portfolio guidelines for submitting code & software for further information.

Data

Policy information about availability of data

All manuscripts must include a data availability statement. This statement should provide the following information, where applicable:

- Accession codes, unique identifiers, or web links for publicly available datasets
- A description of any restrictions on data availability
- For clinical datasets or third party data, please ensure that the statement adheres to our policy

There was no data uploaded on publicly available databases.

Human research participants

Policy information about studies involving human research participants and Sex and Gender in Research.

Reporting on sex and gender	N/A
Population characteristics	N/A
Recruitment	N/A
Ethics oversight	N/A

Note that full information on the approval of the study protocol must also be provided in the manuscript.

Field-specific reporting

Please select the one below that is the best fit for your research. If you are not sure, read the appropriate sections before making your selection.

☒ Life sciences ☐ Behavioural & social sciences ☐ Ecological, evolutionary & environmental sciences

For a reference copy of the document with all sections, see nature.com/documents/nr-reporting-summary-flat.pdf

Life sciences study design

All studies must disclose on these points even when the disclosure is negative.

Sample size	The design and application of sample size were described in the relevant manuscript.
Data exclusions	No data was excluded in analyses.
Replication	The replication was described in the relevant manuscript.
Randomization	The embryos, tadpoles, or frogs used in the same experiment were chosen in random in the same batch.
Blinding	No blinding was applicable.

Reporting for specific materials, systems and methods

We require information from authors about some types of materials, experimental systems and methods used in many studies. Here, indicate whether each material, system or method listed is relevant to your study. If you are not sure if a list item applies to your research, read the appropriate section before selecting a response.

Materials & experimental systems

n/a	Involved in the study	
☐	☒	Antibodies
☒	☐	Eukaryotic cell lines
☒	☐	Palaeontology and archaeology
☐	☒	Animals and other organisms
☒	☐	Clinical data
☒	☐	Dual use research of concern

Methods

n/a	Involved in the study	
☒	☐	ChIP-seq
☒	☐	Flow cytometry
☒	☐	MRI-based neuroimaging

Antibodies

Antibodies used	anti-Tyrosinase (ab180753, Abcam), anti-EGFP (ab184601, Abcam), anti-CD31 (ab5690, Abcam), and anti-ERK1+ERK2 (184699, Abcam) were used in this research.
Validation	These antibodies were used in immunofluorescence applied on paraffin slices of Xenopus tropicalis skin to test the chromatophores in this research. The fluorescence signals were in accordance with the distribution position and shape of chromatophores of Xenopus tropicalis. These results validated the availability of antibodies in Xenopus tropicalis skin, while the detailed results were explained in the relevant manuscript.

Animals and other research organisms

Policy information about studies involving animals; ARRIVE guidelines recommended for reporting animal research, and Sex and Gender in Research

Laboratory animals	Xenopus tropicalis, Xenopus laevis and zebrafish
Wild animals	N/A
Reporting on sex	Both male and female frogs were used in this research. No distinct difference between these two sexes.
Field-collected samples	N/A
Ethics oversight	The ethics were evaluated and approved by Institutional Animal Care and Use Committee at the Southern University of Science and Technology.

Note that full information on the approval of the study protocol must also be provided in the manuscript.
